# The role of language in emotion: predictions from psychological constructionism

**DOI:** 10.3389/fpsyg.2015.00444

**Published:** 2015-04-14

**Authors:** Kristen A. Lindquist, Jennifer K. MacCormack, Holly Shablack

**Affiliations:** Carolina Affective Science Laboratory, Department of Psychology, University of North Carolina, Chapel Hill, NCUSA

**Keywords:** language, emotion, psychological constructionism, concept acquisition, emotional development, concept knowledge, abstract concepts

## Abstract

Common sense suggests that emotions are physical types that have little to do with the words we use to label them. Yet recent psychological constructionist accounts reveal that language is a fundamental element in emotion that is constitutive of both emotion experiences and perceptions. According to the psychological constructionist Conceptual Act Theory (CAT), an instance of emotion occurs when information from one’s body or other people’s bodies is made meaningful in light of the present situation using concept knowledge about emotion. The CAT suggests that language plays a role in emotion because language supports the conceptual knowledge used to make meaning of sensations from the body and world in a given context. In the present paper, we review evidence from developmental and cognitive science to reveal that language scaffolds concept knowledge in humans, helping humans to acquire abstract concepts such as emotion categories across the lifespan. Critically, language later helps individuals use concepts to make meaning of on-going sensory perceptions. Building on this evidence, we outline predictions from a psychological constructionist model of emotion in which language serves as the “glue” for emotion concept knowledge, binding concepts to embodied experiences and in turn shaping the ongoing processing of sensory information from the body and world to create emotional experiences and perceptions.

## Language and Emotion

Common sense suggests that language has naught to do with emotion. Surely, the things that people say affect our emotions, and we can describe our emotions (or the emotions we see in others) with words after the fact. However, it is typically assumed that this is the extent of the relationship between language and emotion. Many contemporary psychological models of emotion agree with this common sense perspective. In these views, emotions are physical types that are essentially distinct from linguistic or conceptual processing (Ekman and Cordaro, 2011; Panksepp, 2011; Shariff and Tracy, 2011; Fontaine et al., 2013). Yet growing psychological research suggests that the role of language may run deeper in emotions than either laypeople or researchers previously thought.

In this paper, we introduce a psychological constructionist model of emotion that explains the mechanisms by which language plays a fundamental role in emotion. We begin our article by first providing a brief primer on the psychological constructionist approach we take in our own work called the Conceptual Act Theory (CAT; cf., Barrett, 2006b). We outline the CAT’s predictions for the role of language in emotion and discuss early evidence that language does indeed play a role in emotion. To understand the ultimate and proximate mechanisms by which language plays a role in emotion, we next explore evidence from developmental and cognitive science, demonstrating that language helps humans acquire and then use concept knowledge to make meaning of their experiences and perceptions. We close by exploring the implications of language’s role in emotion concept acquisition and use for emotional experiences and perceptions.

## Psychological Construction and the Conceptual Act Theory

The idea that language goes beyond describing emotion after the fact is consistent with psychological constructionist theories of emotion. Psychological construction is a family of theories that conceives of emotions as psychological “compounds” resulting from the combination of more basic psychological “elements” that are not themselves specific to emotions (Russell, 2003; Barrett, 2006b, 2013; Clore and Ortony, 2008, 2013; Cunningham et al., 2013; Lindquist, 2013; see Gendron and Barrett, 2009 for a historical account of psychological constructionist views). All constructionist theories of emotion predict that psychological compounds such as anger, disgust, fear, etc. emerge when more basic psychological elements such as representations of the body, exteroceptive sensations (e.g., visual sensations; auditory sensations) and concept knowledge about emotion categories combine. Just as chemical compounds (e.g., NaCl) emerge from more basic elements and possess attributes that their constitutive elements do not—NaCl (sodium chloride, or commonly, table salt) has properties that are not reducible to either sodium, which is a member of the alkali metal family, or chlorine, which is a type of halogenic gas—psychological compounds such as emotions are more than the sum of representations of the body, exteroceptive sensations, and concept knowledge. Most psychological constructionist views agree that a person experiences an emotion when concept knowledge (e.g., knowledge about “fear”) and exteroceptive sensations (e.g., the sights and sounds of being in a dark alley) are used to make meaning of body states (e.g., a beating heart, sweaty palms, and feelings of startle) in a given instance. A person sees someone else as emotional when concept knowledge (e.g., knowledge about “fear”) and exteroceptive sensations (e.g., the sights and sounds of riding a roller coaster) are used to make meaning of someone else’s affective bodily and facial muscle movements (e.g., a person’s wide eyes, gaping mouth, and white knuckles). Our own psychological constructionist approach, the CAT (Barrett, 2006a, 2009, 2012; Wilson-Mendenhall et al., 2011; Lindquist and Barrett, 2012; Lindquist, 2013) specifically predicts a role for language in this process, insofar as language supports the acquisition and use of concept knowledge (e.g., the concept of “fear”) that is used to make sensations meaningful as emotions.

### Basic Elements of the Mind

According to the CAT (CAT; cf., Barrett, 2006a; Lindquist, 2013), the basic elements that contribute to emotions (and other mental states) are representations of sensations from inside the body (known as *affect*), representations of sensations from outside the body (known as *exteroceptive sensations*), and *concept knowledge* used to make those sensations meaningful in context (cf., Barrett, 2009; Lindquist and Barrett, 2012; Lindquist et al., 2012; Lindquist, 2013). Affect is a representation of the body’s ever-changing internal state, which can be experienced as having some degree of valence and arousal or “activation” (Cacioppo et al., 2000; Russell, 2003; Barrett, 2006b; Kober et al., 2008; Mauss and Robinson, 2009; Lindquist et al., 2012; Clore and Ortony, 2013; Cunningham et al., 2013). Affect is often described as a homeostatic barometer that allows an organism to understand whether objects in the world are good for it, bad for it, approachable or avoidable (Barrett and Bliss-Moreau, 2009). Affect is a combination of interoceptive information from the internal milieu that represents activity in the smooth muscles, skeletal muscles, peripheral nervous system, and neurochemical/hormonal system (Barrett and Bliss-Moreau, 2009; Lindquist, 2013). Affect thus provides an internal representation of the meaning of objects in the world and can serve as a “common currency” for comparing the meaning of otherwise diverse stimuli and events (Cabanac, 2002).

By contrast, exteroceptive sensations provide an organism with a representation of information from the external world outside of the body (e.g., vision, audition, taste, olfaction, and proprioception; Barrett, 2009; Lindquist and Barrett, 2012; cf., Lindquist et al., 2012). Exteroceptive sensations contribute to perceptions of emotions in other people (via vision, audition, and perhaps even tactile or olfactory sensations) but are also often the sources of shifts in core affect (e.g., visual sensations of a dark, long, squiggly shape in the middle of the path ahead of you in the woods) that contribute to one’s experiences of emotions in his or her own body and provide information about the physical context that is used to help disambiguate the meaning of interoceptive sensations.

Importantly, the CAT predicts that both affect and exteroceptive sensations are made meaningful as instances of specific emotional experiences or perceptions using concept knowledge about emotion categories (Barrett, 2006a, 2009, 2014; Lindquist, 2013; also see Russell, 2003; Clore and Ortony, 2013; Cunningham et al., 2013 for other psychological construcitonist views). Concept knowledge refers to the rich cache of instances that populate what someone “knows” about different categories. For instance, people may know that the category of *fear* involves a beating heart, sweaty palms, a knot in the stomach, an urge to flee, and threatening contexts related to various objects (e.g., snakes, bears, cliffs, intruders, etc.). Notably, people also know lots of other information about fear, even if it’s not stereotypical of fear, and this information may vary ideographically—for instance, one person might know that fear can involve attacking someone else; another person might know that fear can involve smiling. Still other people might know that fear can variably involve clowns, global warming, public humiliation, and existential concerns. Rather than consisting of a number of prototypes for certain emotions, concept knowledge about emotion is thus thought to consist of populations of instances (cf., Barrett, 2012) that have been acquired via a combination of instrumental learning via other individuals (i.e., “semantic knowledge”) and personal experience (i.e., “episodic knowledge”; see discussion in Vigliocco et al., 2009).

Once acquired, concept knowledge serves as a form of *a priori* information to shape predictions about new interoceptive and exteroceptive sensations, helping the brain understand the meaning of sensations and act on them (Bar, 2007; Barrett, 2009, 2014; Clark, 2013). In the case of emotion, this means that concept knowledge is used to help make otherwise vague and potentially ambiguous sensations from inside the body (affect) and outside the body (exteroceptive sensations) meaningful as instances of specific emotions (e.g., anger, disgust, fear, pride, joy, schadenfreude, what have you). The resulting emotion is thus an emergent state that is at once affective and conceptual (cf., Lindquist and Barrett, 2008a). We refer to the process of using knowledge to make meaning of sensations as *situated conceptualization*, because the concept knowledge accessed to make meaning of sensations is highly situated and dependent on the present context. Situated conceptualization is a relatively automatic^1^ process (Wilson-Mendenhall et al., 2011; Barrett, 2014) and operates in a probabilistic manner (Barrett et al., 2007b; Clark, 2013), making predictions about the meaning of sensations (e.g., a beating heart, sweaty palms) given the features of the present context (e.g., giving a speech), prior experiences of other contexts in which similar sensations have occurred (e.g., past experiences of giving speeches vs. past experiences of watching scary movies vs. experiences of standing atop a tall balcony), and culturally relative knowledge about the types of experiences that involve certain sensations (e.g., knowledge about fear vs. excitement).

Importantly, the CAT predicts that the aforementioned elements are domain-general elements of the mind and are not specific to the category of mental states called “emotions” (Barrett, 2009; Lindquist and Barrett, 2012; Barrett and Satpute, 2013; Lindquist, 2013). In essence, the CAT does not see “emotions” as states that are fundamentally distinct from “cognitions” or “perceptions” (cf., Barrett, 2009; Lindquist, 2013; e.g., Oosterwijk et al., 2012); all are constructed from the same basic elements and are *nominal kind* categories that exist because members of a culture agree that they share certain features (e.g., in English, “emotions” are typically thought to involve relatively greater involvement of the body than “thoughts,” even if body states are in fact constitutive of both kinds of mental states; e.g., Oosterwijk et al., 2012). The agreement between members of a culture imbues emotions with social reality—they are real even if the specific categories (e.g., anger, disgust, fear, sadness, schadenfreude, pride, excitement, awe, etc.) are not inborn categories given by the structure of the nervous system (cf., Barrett, 2012). In this sense, the CAT and other constructionist views are quite distinct from other psychological and neuroscience models of emotion, which view emotions as domain-specific, inborn, inherited types that are fundamentally distinct from other types of mental states (e.g., “cognitions,” “perceptions,”), and are produced by specific anatomically-given neural structures (i.e., emotions are *natural kind* categories; e.g., Cannon, 1921; Allport, 1924; Tomkins, 1962; Izard, 1971; Sprengelmeyer et al., 1996; Ekman and Cordaro, 2011; see Barrett, 2006b for a review)^2^. In such natural kind views, there is no role for language in the constitution of emotion (Ekman and Cordaro, 2011; Panksepp, 2011; Shariff and Tracy, 2011; Fontaine et al., 2013) and the role of language in the acquisition of emotion concepts should have no bearing on the actual experience or perception of emotion. The predictions of the CAT are thus quite novel in regard to emotions, even if they are more broadly consistent with other evidence that language generally supports the construction of “cognitive” mental states (e.g., Boroditsky, 2011; Lupyan, 2012a,b,c).

### Growing Evidence: A Role for Language in Emotion

In contrast to the natural kind view of emotion, there is growing evidence for the CAT’s prediction that concept knowledge supported by language plays a constitutive role in emotions. In recent years, we have extensively reviewed the literature on language and emotion (Barrett et al., 2007a; Lindquist and Gendron, 2013; Lindquist et al., in press a,b) documenting the various ways in which language shapes on-going perceptions and experiences of affect into perceptions and experiences of emotion (anger, disgust, fear, sadness, etc.). For instance, we have documented that impairing people’s access to the meaning of emotion words impairs their ability to subsequently perceive emotions on faces (Lindquist et al., 2006, 2014; Gendron et al., 2012). Without access to the meaning of emotion words such as “disgust,” vs. “anger,” vs. “fear,” vs. “sadness,” individuals perceive posed emotional facial expressions (wrinkled noses, scowls, wide eyes, and frowns) as merely unpleasant (Lindquist et al., 2014). These findings suggest that access to the meaning of emotion words (and the concepts that they represent) is an essential component of understanding the discrete meaning of emotional facial expressions.

Other research demonstrates that labeling one’s own unpleasant feelings with emotion words causes an experience of a particular discrete emotion to occur. Individuals who are exposed to labels for the category “fear” prior to listening to unpleasant music are subsequently more likely to engage in behaviors typical of fear (i.e., risk aversion) than individuals who were exposed to labels for the category “anger” or those not exposed to emotion category labels at all prior to listening to unpleasant music (Lindquist and Barrett, 2008a). Labeling one’s affective state as an emotion also alters cardiac responses during affective events. Individuals who labeled their emotions while completing a stressful mental arithmetic task showed physiological responses consistent with an experience of threat (i.e., increased total peripheral resistance or TPR; relatively reduced cardiac output), whereas participants who did not label their emotions experienced a physiological profile more consistent with active coping (i.e., decreased TPR, increased cardiac output; Kassam and Mendes, 2013). These findings suggest that labeling an unpleasant state as one type of emotional experience vs. another can shape how it is subsequently experienced.

Neuroscience evidence also documents a critical link between language and emotion. Growing evidence suggests that using emotion words to label posed emotional facial expressions reduces activity in brain regions associated with uncertainty such as the amygdala (Lieberman et al., 2007; see Lindquist et al., in press b for a discussion). These findings are consistent with the idea that emotion words help to make meaning of otherwise ambiguous unpleasant vs. pleasant facial expressions (cf., Lindquist et al., in press b). Consistent with the interpretation that language plays a routine role in creating instances of discrete emotion perceptions and experiences, meta-analytic summaries of the neuroimaging literature on emotion reveal that a subset of the brain regions involved during studies of emotion perceptions and experiences are also involved during studies of semantic judgments (Lindquist et al., in press b). Together, these accumulating sources of evidence suggest that language may not merely impact emotions after the fact. They instead suggest that language plays an integral role in emotion perceptions and experiences, shaping the nature of the emotion that is perceived or felt in the first place.

Finally, evidence from cross-cultural research is consistent with the idea that language plays a constitutive role in emotion. For instance, speakers of Herero, a dialect spoken by the remote Himba tribe in Namibia, Africa, and American English speakers perceive emotions differently on faces. When participants were asked to freely sort images of identities making six facial expressions (anger, disgust, fear, happiness, sadness, and neutral) into piles, English-speakers created relatively distinct piles for anger, disgust, fear, sad, happy and neutral faces, but Herero-speakers did not sort in this pattern. Instead Herero-speakers produced piles that reflected multiple categories of facial expressions (e.g., smiling, neutral, wrinkled nose, scowling, and frowning faces). Importantly, the Herero-speakers sorted similarly to one another, suggesting that they understood the instructions but were using different perceptual cues (and perhaps different categories) than the English-speakers to guide their sorts (Gendron et al., 2014).

The existing evidence thus suggests that language plays some role in emotion, but what remains in question is the precise mechanisms by which language does so. The CAT hypothesizes that language helps support the acquisition and use of concept knowledge about emotion, but very little work has directly addressed this hypothesis in relation to emotion, to date. We thus turn now to evidence from developmental and cognitive science demonstrating that language helps individuals represent and use concept knowledge in general, as well as concept knowledge about emotions in particular. We use this evidence to hypothesize about the mechanisms by which language shapes the acquisition and subsequent use of emotion concept knowledge.

## Language Supports Conceptual Knowledge of Emotion

The CAT makes the unique prediction that language plays a role in emotion because language helps a person to initially acquire and then later support the representations that comprise emotion concept knowledge (Lindquist, 2013; cf., Lindquist et al., in press b). Of course, language likely plays a role in the acquisition and use of all category knowledge (see Lupyan, 2012a,b; Borghi and Binkofski, 2014). However, we hypothesize that language is especially likely to be implicated in emotion because emotion concepts (e.g., *anger, disgust, fear*, etc.) are embodied and abstract representations that form populations of conceptual information rather than concrete concepts grounded by physical types that form prototypes for emotion category knowledge. Words for emotion categories (e.g., “anger,” “disgust,” “fear”) thus serve as the “glue” or “essence place-holder” (cf., Xu, 2002) that helps bind together otherwise disparate instances of a given emotion category^3^.

The idea that emotion concepts are embodied derives from growing evidence in cognitive science that conceptual knowledge is represented via sensorimotor “simulations” of prior sensory experiences and actions (Glenberg and Gallese, 2012; for review, see Kiefer and Barsalou, 2013). Traditionally, researchers assumed that emotion concepts are structured as prototypes (Shaver et al., 1987; Russell, 1991) or as theories about why category members share certain features (Clore and Ortony, 1991; Zinck and Newen, 2007). In these models, category knowledge is represented outside the sensory modalities as amodal, symbolic representations (see Barsalou, 1999). Instead, consistent with recent theories of embodied cognition (e.g., Barsalou, 2009; Vigliocco et al., 2009; Borghi and Binkofski, 2014), the CAT proposes that prior perceptual experiences associated with an emotion category help constitute conceptual knowledge of that emotion. Emotion categories are thus represented as re-enactments of prior interoceptive sensations such as feelings (see Barrett and Lindquist, 2008; Wilson-Mendenhall et al., 2013a), modality-specific exteroceptive perceptions such as visual, auditory, olfactory, and proprioceptive sensations of objects and contexts (e.g., Wilson-Mendenhall et al., 2011, 2013b), and actions (e.g., punching vs. yelling vs. scowling vs. smiling in anger; Oosterwijk et al., 2014; see Barrett and Lindquist, 2008 for a discussion). Emotion concepts also contain more abstract, propositional information that describes a person’s relationship to the environment (e.g., sadness is about loss); this information may be acquired from one’s culture and augmented by prior experiences (i.e., representations of specific instances of loss). For example, the concept of what it feels like to be “sad” may include previous bodily sensations (e.g., feeling heavy, drained, tired; unpleasant), previous exteroceptive sensations (i.e., sights, smells, tastes, sounds, associated with different physical contexts in which one was sad), and simulations of representative instances in which loss occurred (e.g., simulations of the context in which loss occurred at the death of a loved one, during an insult to one’s self-esteem, loss of a job, etc.).

Based on evidence that the bodily and exteroceptive concomitants of instances of a single emotion category are highly variable (Cacioppo et al., 2000; Barrett, 2006b; Mauss and Robinson, 2009; Kreibig, 2010), the CAT also proposes that embodied emotion categories are abstract—without a single category prototype to define them (for a similar view, see Vigliocco et al., 2009; Borghi and Binkofski, 2014). In this view, emotions are not natural kind categories with strong perceptual regularities (Barrett, 2006a), nor are they single prototypes that stand in as typical examples of the rest of the category members (Barrett, 2014). Unlike concrete categories (e.g., “apple”) that may have strong perceptual regularities (e.g., apples are round, tart, crisp, red/green/yellow fruits that grow on trees) and clear, best example prototypes (e.g., a Red Delicious), emotion categories are thought to exist as *populations* of conceptual information that might not be covered by a single “best example” category prototype (Barrett, 2014). In this view, there are many sensorimotor representations of “anger” that help form conceptual knowledge about this category and there is little perceptual regularity that makes instances obviously similar to one another (e.g., not all instances of anger involve a scowl, increased heart rate, punching, etc.).

Research demonstrates that situations may be key for an individual to acquire abstract concepts, such as “anger,” “love,” “fear,” or “pride,” that do not correspond to strong statistical regularities in exteroceptive or interoceptive sensations. For example, when individuals are asked to think about the abstract concepts “convince” and “arithmetic,” brain regions associated with contexts in which those concepts might be involved are activated (Wilson-Mendenhall et al., 2013a). When thinking about the word “convince,” brain regions associated with mentalizing and social cognition are activated. By contrast, when thinking about arithmetic, brain regions associated with engaging in numerical cognition are activated. Similarly, representations of emotion concepts draw on situations, with representations of fear involving brain networks underlying different types of contexts (Wilson-Mendenhall et al., 2013b). In some instances, brain regions involved in representing fear include those involved in social inference and mentalizing (i.e., as might occur during social threats). By contrast, other representations of fear involve networks underlying visuospatial attention and action planning (i.e., as might be observed during physical threats; Wilson-Mendenhall et al., 2013b). Abstract concepts can thus be thought of as reconstituted amalgamations of situated experience, and these amalgamations evolve with new experiences and new information from early life across the lifespan (Meteyard et al., 2012). Replaying or “simulating” the situation in which an abstract concept occurs may in part be what enables individuals to use abstract concepts to make future situated conceptualizations. Consistent with this hypothesis, research finds that lexical access, word comprehension, and memory are generally faster for concrete concepts than abstract ones; however, when situational cues are provided, abstract concepts become just as quickly available as concrete concepts (Barsalou and Wiemer-Hastings, 2005).

Critically to this paper, another key to acquiring and using abstract concepts such as emotion concepts may be language (cf., Barrett and Lindquist, 2008; Borghi and Binkofski, 2014). An embodied theory of language and semantics assumes that the brain’s linguistic system is separate from, but integrally tied to the modality-based system that represents embodied concepts (Barsalou and Wiemer-Hastings, 2005; Barsalou et al., 2008; Vigliocco et al., 2009). In the absence of strong statistical regularities based on previous perceptions of concrete objects in the environment, abstract concepts may particularly benefit from language—that is from being associated with the phonological form of a word (Barrett and Lindquist, 2008; Vigliocco et al., 2009; Borghi and Binkofski, 2014). People may integrate in long-term memory two representations from the same emotion category (even if they involve different bodily and exteroceptive sensations, contexts, and actions) because the label for the emotion links them in memory (see Gelman and Markman, 1987; Borghi and Binkofski, 2014). As foreshadowed by early researcher Hunt (1941, p. 266), the CAT predicts that, “the…universal element in any emotional situation is the use by all the subjects of a common term of report (i.e., ‘fear’).”

The CAT of emotion thus predicts that language plays a role in emotion because it helps individuals to initially acquire and then use emotion concept knowledge to form situated conceptualizations of affect (for a similar view of abstract concepts, see Borghi and Binkofski, 2014). To date, research assessing the CAT has focused exclusively on documenting evidence that language plays a role in emotion experiences and perceptions at all. However, very little research to date has addressed whether language specifically helps individuals acquire and use words to make situated conceptualizations of emotion across development, which might form the ultimate mechanisms by which language shapes emotion. Growing evidence from developmental and cognitive science demonstrates that words help infants and adults acquire and then use concepts throughout the lifespan; this evidence suggests that language is key to the acquisition of emotion concepts. We thus turn to this literature to motivate predictions for how words help individuals acquire the emotion concepts that they then use to make situated conceptualizations about emotion.

## Words Help Humans Acquire Emotion Concepts: Lessons from Early Development and Adult Cognition

### Language and the Acquisition of Emotion Concept Knowledge in Infants

Understanding how infants and young children use words to learn novel concepts sheds light on how language more generally contributes to the acquisition of concept knowledge, and by extension, concept knowledge about emotion. Developmental accounts of concept knowledge traditionally assumed that infants are either born with pre-existing knowledge of specific categories (a nativist account) or learn every category *de novo* (an empiricist account; for discussion, see Xu and Griffiths, 2011). Similarly, before the recent emergence of constructionist accounts of emotion, many models of emotion assumed that infants were born with the ability to experience and perceive basic emotions such as fear, sadness, and disgust (Izard, 1978, 2007, 2011; Ekman and Oster, 1979; Barrera and Maurer, 1981; Campos et al., 1992; Lewis, 2000). However, growing research suggests that infants are “rational constructivists,” born without pre-existing knowledge of many categories, but possessing an intrinsic sensitivity to statistical regularities and the ability to extrapolate to new category instances on the basis of inductive learning (Sirois et al., 2008; Xu and Kushnir, 2013)^4^. The ability to use statistical regularities extracted from prior experience to categorize phenomena and predict those phenomena’s causes, behaviors, and effects is known as probabilistic or statistical learning. Probabilistic learning continues across the lifespan and is a fundamental aspect of human cognition (Oaksford and Chater, 2009; Carey, 2011; Clark, 2013). We propose that this basic ability also undergirds infants’ abilities to learn about emotion categories through words.

From the early days of brain development *in utero*, infants’ brains are able to observe stimuli from inside and outside their bodies and begin forming probabilistic *a priori* predictions about the meaning of those stimuli (Aslin and Newport, 2012). For instance, probabilistic learning may first exert its influence when infants learn to categorize sounds as linguistic vs. non-linguistic *in utero*. Organization of the auditory cortex in humans is thought to occur by the 27th week of gestation (Hepper and Shahidullah, 1994) and plasticity in the auditory cortex is thought to occur due to sounds penetrating the mother’s intrauterine walls (Gerhardt and Abrams, 2000). Fetuses exposed to particular phonemes (linguistic speech sounds) *in utero* show more neural responsiveness to those phonemes after birth than newborns that were not exposed to such phonemes as fetuses (Partanen et al., 2013). This early sensitivity to language suggests that even as neonates, infants bring with them the ability to differentiate and make predictions about different linguistically relevant sounds. Indeed, neonates who are less than a day old already prefer phonemes from their native language to phonemes from a non-native language (Moon et al., 2013). After birth, infants use the statistical properties of language to help them differentiate between phonemes and extrapolate rules of grammar in their native language (Saffran et al., 1996; Aslin et al., 1998; Maye et al., 2002; Thiessen and Saffran, 2003; Kuhl, 2004; Rivera-Gaxiola et al., 2005; Gebhart et al., 2009; Teinonen et al., 2009; Shukla et al., 2011; Krogh et al., 2013).

Just as infants use statistical learning to differentiate words from non-words, they also use probabilistic learning to understand visual sensations in the world around them; these two processes likely co-occur and interact (for review, see Bergelson and Swingley, 2012). By 3–4 months of age, infants begin to form categories for natural kinds (e.g., species categorization for horses, zebras, tigers, cats, etc.; see Eimas and Miller, 1992; Quinn and Eimas, 1996) and artifacts (e.g., different furniture types such as chairs, tables, and beds; Behl-Chadha, 1996). Some concept knowledge for these categories may be developed on the basis of visual statistical regularities alone (e.g., all zebras have stripes, horses do not). Yet not all categories can be learned on the basis of statistical regularities alone, especially abstract categories, and so it is predicted that infants use the phonological sound of a word as a salient cue for differentiating between sensations in the environment. This is particularly relevant for emotion categories, where the word “anger” for example can tie together multiple modalities of sensorimotor experience (such as bodily sensations, situations, or behaviors) and also can serve as “glue” for different instances of “anger” that are not necessarily perceptually regular or consistent with one another (e.g., being angry at one’s computer may not look or feel the same as being angry about an insult).

Thus, by 9 months of age, infants regularly use words as cues for understanding which objects in the world are similar vs. distinct. For example, the presence of two distinct labels helps infants establish a representation that two objects are in fact distinct in an object individuation task (Xu, 2002). Words seem to be special in this regard; the presence of two different labels facilitates object individuation, but the presence of two distinct tones, two distinct sounds, or two distinct emotional expressions does not^5^. By contrast, when 9-month-old infants hear one label repeated twice, they expect to see two objects that are perceptually similar (Dewar and Xu, 2009). By around a year of age, infants can use the presence of words to make predictions about the types of stimuli to expect. Twelve-month-old infants will look for two objects when an adult uses two words as opposed to one word to describe objects that are unseen by the infant (Xu et al., 2005). Similarly, emotion labels may be an important cue for helping infants and young children understand emotion categories and apply those categories to their own experiences and observations.

Importantly, for abstract concepts that do not have strong perceptual similarities, labels also help infants learn that perceptually distinct objects should be treated as members of the same category. For instance, in 10-month-olds, linguistic labels can override the perceptual qualities of objects, directing infants to group together objects that do not possess strong perceptual similarities (Plunkett et al., 2008). When infants are taught to group cartoon creatures possessing various features (e.g., differences in tail size, head size, etc.) into categories, the use of a single label leads infants to sum across perceptual differences within the cartoons and learn a single category that included all the cartoon creatures. Thus, words not only inform infants about the nature of phenomena they encounter and help them classify what phenomena go together, but words also tell children where to look for boundaries between categories—including categories that might not be perceptually obvious but that are encoded in language (Bowerman, 1988; Roberts and Jacob, 1991). No research to date has directly examined this hypothesis with the acquisition of emotion category knowledge in infancy, but we predict that infants may be using words to help derive emotion categories to describe affective sensations in their own bodies and expressions of affect seen in others’ bodies.

### Language and the Acquisition of Emotion Concept Knowledge in Young Children

Once infants become verbal toddlers, their concepts become honed through bi-directional communication with caregivers. As infants begin producing words themselves, they have the opportunity to receive more directed feedback from adults as to whether their word-sensation associations map on to the word-sensation associations of adults in their culture. Research from computer simulations suggest that the communicative function of language may be essential for helping humans to develop concept knowledge that is shared with other societal members. For instance, Steels and Belpaeme (2005) programmed artificial intelligence agents in a simulation to each possess the same capabilities for perception, categorization, and naming of colors in the artificial environment. The color space in this artificial environment was a set of continuous wavelengths of light with no statistical regularities in terms of the contexts in which certain color categories appear. Each agent was furthermore programmed to experientially develop its own unique knowledge of which sensory information corresponded to which categories and words. In one simulation, the agents merely learned to discriminate a given color from the present sensory array (all of color space) and named the color based on their personal set of category representations. Yet in a separate simulation, the agents not only discriminated for themselves but also “communicated” with one another, allowing each agent to learn category knowledge from the present social interaction in which they were involved. In this social interaction, the first agent (the speaker) discriminated a given color from the present sensory array (all of color space) and named the color based on the agent’s own set of personal color category knowledge. The second agent (the hearer) then had to guess which color the speaker was referring to. If the hearer was successful, it strengthened the association between the word used and its own personal color category knowledge. Yet if the hearer was unsuccessful, it lessened the association between the word used and its own color category knowledge and also created a new association between the word the speaker used and color category knowledge. The authors found that although agents in the first scenario learned to discriminate between different colors and each developed their own set of color category knowledge from the environment, each agent possessed completely different color knowledge when the simulation was over. By contrast, in the simulation involving communication, all agents eventually possessed the same color knowledge. Importantly, similar results persisted even when statistical regularities were introduced in terms of which colors occurred in which contexts in the artificial environment (a situation that likely better approximates the real world). By extension, these findings suggest that children might never learn the emotion concepts of their culture without communication with caregivers.

In light of the importance of communication in concept acquisition, it is interesting that children do not learn how to reliably categorize facial expressions of different emotions (e.g., “anger,” “disgust,” “fear,” “sadness”) as distinct until they acquire and begin to use words to describe those categories in conversation. Although there is debate on this point, pre-linguistic infants and toddlers younger than 2 years of age seem only able to reliably differentiate facial expressions in terms of valence (i.e., positivity vs. negativity; for reviews see Widen and Russell, 2008; Widen, 2013)^6^. Two-year-olds use the very simple emotion labels “angry” and “happy” in daily discourse and, like infants, can reliably differentiate faces in terms of valence. Yet 2-year-olds cannot differentiate between more specific unpleasant emotion categories until they start reliably using additional negative emotion terms in daily discourse (Widen and Russell, 2008). For example, when 2-year-olds are given a set of pictures depicting five emotion categories and are asked to perceptually match only those faces that match an additional picture (e.g., an angry face) by placing them in a box, they place all unpleasant faces (angry, sad, disgusted, fearful faces) in the box but leave out happy faces. Yet as 3- and 4-year-olds begin to acquire the concepts “sad” and “fear,” they begin to leave those faces out of the “angry” box, demonstrating an ability to perceptually categorize unpleasant faces into more specific emotions. By the age of 7, children show adult-like perceptual categorization of most faces save disgust (Widen and Russell, 2008; see Widen, 2013 for a review). These findings suggest that as children acquire emotion words and start using them in daily life with caregivers, they become increasingly competent at perceiving and labeling facial expressions in terms of their culture’s emotion categories. Consistent with the idea that words help infants generalize between otherwise perceptually distinct objects during learning, toddlers appear to show a “language superiority effect” when categorizing facial expressions (Russell and Widen, 2002). Specifically, 2- and 3-year-olds are better able to accurately place pictures of facial expressions in a box labeled with a word (e.g., “anger”) as compared to a box labeled with a face (e.g., an angry face), an effect that increases over early childhood. These findings suggest that newly acquired emotion knowledge associated with a word anchor may help children gloss over perceptual similarities between faces that are not useful for the categorization of facial expressions (e.g., furrowed brows in both anger and disgust) and focus on perceptual differences that are diagnostic (e.g., a scrunched nose in disgust vs. a growl in anger). Such a link between children’s emotion understanding and linguistic development is also suggested in correlational studies demonstrating that children’s advances in emotion understanding develop in tandem with advances in language comprehension (Harris et al., 2005).

We thus predict that emotion concept knowledge acquisition expands as young children acquire words for specific emotions and receive feedback on their situated conceptualizations of their own and others’ affective states through communication with caregivers. Indeed, much evidence is consistent with the idea that communication with parents about emotions during early childhood is essential for children to develop complex knowledge about the emotion categories relevant to their culture (for discussion, see Halberstadt and Lozada, 2011). The implication of these findings is that parents’ own abilities at situated conceptualization, concept knowledge about emotions, and communication skills, can transfer to their children. For instance, 2–4 year old children’s total emotion utterances correlate with the emotion labels that their mothers know and use (Cervantes and Callanan, 1998). Similarly, children whose mothers used more emotion terms when children were 18 months old in turn produced more emotion terms themselves at 24 months (Dunn et al., 1987). Children whose parents discussed emotions more when children were 36 months old also had better emotion understanding at 6 years of age (Dunn et al., 1991). Parents’ explanations of internal states and attributes (such as “hungry,” “sad,” or “nice”) are thus thought to scaffold children’s own abilities to identify and describe the same experiences in themselves and others (Saarni, 1999; Yehuda, 2005), perhaps because word use is helping children acquire complex embodied information about a given emotion category.

By contrast, parents who possess a paucity of conceptual knowledge about emotion or who struggle to communicate this knowledge likely dampen their children’s opportunities to develop conceptual knowledge about emotion. Alexithymia is a non-clinical characteristic commonly defined as “difficulty identifying, understanding, and expressing feelings” (Bagby et al., 1986) and is hypothesized to stem in part from a paucity in conceptual knowledge about emotions (cf., Lindquist and Barrett, 2008b). In this view, adults with alexithymia either possess relatively sparse knowledge about specific emotion concepts (e.g., knowledge about *fear* might consist of a relatively narrow population of instances) or do not have differentiated knowledge about emotion concepts in the first place (e.g., these individuals do not possess differentiated concepts for *anger*, *disgust*, and *fear* and instead just possess a concept for *negativity*). The result is that they themselves have difficulty making situated conceptualizations of affective states in the moment, which in turn limits their ability to translate this knowledge to their children. Consistent with this interpretation, there is some evidence that the tendency for alexithymia is transmitted across generations; caregivers who struggle to communicate and express their feelings create an impoverished environment for children to learn conceptual knowledge about emotions (Berenbaum and James, 1994; Lumley et al., 1996). For instance, college students’ level of alexithymia is positively correlated with their mothers’ retrospective difficulty expressing feelings when their children were young (Fukunishi and Paris, 2001).

Similarly, evidence suggests that parents’ beliefs about emotions, which can be considered a meta-cognitive aspect of emotion concept knowledge, shape children’s emotional abilities. Parents’ beliefs about the value of emotions guides both how parents talk about emotions to children, but also how parents react to their children’s emotions (Dunsmore and Halberstadt, 1997; Hakim-Larson et al., 2006). For example, parents who believe that emotions are valuable are more likely to discuss and teach children about emotions (Gottman et al., 1996); this in turn gives children an opportunity to discuss their growing conceptual knowledge about different emotions and get feedback on the situated conceptualizations they are making about their and others’ internal states and behaviors. Such exchanges consequently shape children’s socioemotional abilities. For example, one recent study found that parents’ beliefs that emotions are valuable as opposed to dangerous predicted children’s ability to recognize their parents’ emotional facial expressions (Castro et al., 2014). This may be in part because parents who believe that emotions are dangerous are more likely to avoid expressing emotions, creating a more impoverished affective environment for their children to practice their developing emotion-relevant skills (Dunsmore et al., 2009). Another possibility, however, is that parents who avoid talking about emotions to their children due to a belief that emotions are dangerous do not help children acquire the conceptual knowledge necessary for learning how to differentiate between different emotional facial expressions.

Together, the developmental evidence suggests that parents help children acquire emotion concepts, in part through the communicative powers of language, and that parents also may scaffold children through the process of making situated conceptualizations of emotion. Parents constantly infer what they believe their young child may be feeling—based on conceptualizations of their own previous and current interoceptive and exteroceptive experiences, in context of the current situation, their knowledge of how their child usually acts, and how the child is currently behaving. For instance, a father may categorize his preverbal daughter’s internal state as “mad” when he observes her refusing to eat and throwing her food—based on the present context and also based on his knowledge of the contexts in which he experiences frustration himself. He may label her inferred state for her, asking why she is “mad”; this parental labeling may in turn help the child associate her current feelings of unpleasantness, her behaviors, and her father’s reactions in that moment to the word “mad.” Over the course of early childhood, parents (with varying degrees of skill) discuss with their children why the child behaved and felt the way she/he did (“Why were you angry with Grandma?”) and why other people behaved and felt the way they did (“Your friend hit you because he was angry you took his toy”). As children acquire emotion concept knowledge and become able to label their own states, they can receive feedback from adults on the “accuracy” of their situated conceptualizations (e.g., the child reports that she is “sad” because her brother took her toy and a parent corrects that she is more likely to be “mad” that the toy was taken). Over time, with the development of conceptual knowledge and the ability to draw complex inferences about their own and others’ mental states, children’s tendency to make situated conceptualizations of emotion are likely to become more automatic. Ultimately, children whose emotion knowledge is more defined (due to the content communicated by parents) and more automatically accessible (due to motivation to categorize states as emotional instilled by parents) would be more emotionally aware and able to understand the complexities and nuance of emotions in different situations. The benefit of this ability is clear: children who are more skilled at recognizing and expressing their own emotions exhibit less worry and depression than children who struggle to convey their emotional experiences (Rieffe et al., 2007). Likewise, children’s emotion understanding is predictive of their social and emotion regulation skills, as well as their academic outcomes (for reviews, see Halberstadt et al., 2001, 2013).

Thus far, we have discussed concept acquisition as if it halts after early childhood and remains stable thereafter. To the contrary, findings in adults suggest that language may still play a role in adult emotion because language continues to help adults acquire and use concept knowledge to make meaning of core affect and exteroceptive sensations. In fact, a small body of evidence suggests that words help adults learn that novel perceptual instances are either similar or distinct and assists adults in continuing to assimilate new perceptual instances into existing category knowledge. We now turn to this evidence.

### Language and the Acquisition of Concept Knowledge in Adults

In an embodied account of concept knowledge, adults continue to update and refine categories based on on-going experiences of the perceptual world throughout their life (Schyns et al., 1998; Vigliocco et al., 2009; Barsalou, 2012). Growing evidence suggests that words play as much, if not more, of a role in adults’ acquisition of novel visual categories, even when words are redundant with other cues for learning. For instance, in one study documenting the role of language in adult category learning (Lupyan et al., 2007), participants learned to categorize novel “alien” stimuli as things to be approached or things to be avoided and received feedback on the accuracy of each response. As participants received feedback about the accuracy of their judgment, participants in the label condition also saw a nonsense word; participants in the control condition received no word. Even though words were not necessary for the task, those participants who saw nonsense words while learning to categorize the stimuli were later better able to differentiate between members of different categories than were individuals who did not. Redundant words facilitated learning regardless of whether they were presented visually or played aurally during learning.

Despite research on the role of words in general adult concept acquisition, very little work has specifically assessed how words help adults learn novel emotion concepts. Indeed, it is hard to conduct this research because most healthy adults (who are not alexithymic) already possess substantial knowledge about the feelings, situations, behaviors, and bodily changes that accompany the emotion categories encoded by their acquired language. However, one study addressed the role of language in the perception of emotion in a category-learning task involving novel Chimpanzee affective facial actions that were unfamiliar to most participants (Fugate et al., 2010). In the first phase of the experiment adults simply viewed pictures of unfamiliar Chimpanzee facial actions (e.g., a “bared teeth” or “scream” face) or viewed the faces while learning to associate them with nonsense words. Participants were later shown two images taken from a continuous morphed array of two facial expressions (e.g., an image of a face containing a percentage of both the bared teeth expression and scream expression) and were asked to indicate whether two faces from random points throughout the array were similar to one another or different. This was a classic measure of “categorical perception” (Goldstone, 1994), the ability to perceive categories within a continuous dimension of sensory information. On some trials, participants compared faces that did not cross one of the learned category boundaries (e.g., they compared an 86% bared teeth, 14% scream expression with a 71% bared teeth, 29% scream expression), whereas on others, they compared faces that *did* cross a learned category boundary (e.g., compared a 43% bared teeth, 57% scream expression with a 29% bared teeth, 71% scream expression). If participants demonstrated categorical perception, they would see the first set of faces as similar but the second set of faces as different. Yet only participants who learned to associate the faces with words in the first phase of the experiment demonstrated such categorical perception. Participants who did not learn to associate faces with a label did not perceive a categorical distinction between the faces.

Building on these findings, a recent study from our laboratory suggests that language can even help adults acquire and assimilate new perceptual experiences into existing category knowledge about emotional facial expressions (Doyle and Lindquist, in preparation). During a learning phase, participants saw a series of non-stereotypical posed facial expressions of anger (e.g., a scowl and squinted eyes with raised eyebrows) and fear (e.g., an open mouth and wide eyes with furrowed eyebrows). In one between-subjects condition, participants learned to associate these facial expressions with emotion words (“anger” vs. “fear”). In another, participants studied the faces and performed perceptual judgments (whether the eyes were close together vs. far apart). In a target phase, participants next studied target individuals who were depicting stereotypical facial actions for either anger or fear and were asked to categorize the facial expression as “anger” or “fear.” During a final test phase, participants were asked to identify which face the target individual had been making during the target phase (i.e., either the learned face, the target face, or a morphed combination of the two). Consistent with the idea that language helps adults acquire and assimilate new perceptual instances into existing category knowledge, participants who had paired faces with words in the learning phase were more likely to remember seeing a target face that was similar to the learned category information. These findings suggest that language helps acquire novel category knowledge that biases memory of later novel faces.

Together, these early findings point to the idea that language continues to help adults acquire novel category knowledge across the lifespan and to update existing category knowledge. This may be how adults continue to augment their existing category knowledge about emotion and suggests that at any point in time, adults’ category knowledge about emotion may reflect the regularities present in the local environment (e.g., one’s cultural, social, or familial context). For example, if concept knowledge is always being updated and changed, then an adult’s knowledge about say, *anger*, may be impacted by the last time the person experienced an instance of *anger* (e.g., at a spouse). This concept knowledge may thus feed-forward to impact situated conceptualizations of future instances of body states when with a spouse, potentiating the situated conceptualization of *anger* over, say, *anxiety* or even other body states such as *hunger* (e.g., a person might conceptualize her unpleasant feelings around dinner time as *anger* toward her spouse as opposed to *hunger* for the impending meal). Thus, the CAT predicts that language does more than just help acquire concept knowledge. It further predicts that language supports the accessibility and use of existing concept knowledge as humans make meaning of sensations in the body or world during the construction of emotions. This prediction is consistent with growing evidence from cognitive science that language, once connected to certain perceptual representations that become stored as conceptual knowledge, alters on-going adult perception by selecting certain sensations for conscious awareness while suppressing other sensations from conscious awareness.

## The “Label-Feedback Hypothesis”: Language Supports the Use of Concept Knowledge

As we stated earlier, in the field of emotion, the CAT’s prediction about the role of language in emotion are quite novel. However, current evidence in cognitive science converges on the idea that language shapes on-going conceptual processes in adults more generally; these conceptual processes furthermore shape the online processing of external sensations across modalities. According to Lupyan’s (2012a) “label-feedback hypothesis,” labels connected to concepts shape the conceptual information that is brought to bear when making meaning of sensations in the environment (see Figure 1 in Lupyan, 2012b). The label-feedback hypothesis thus explains why language shapes on-going perception (e.g., visual perception) and cognition (e.g., thought) in adults (see Lupyan, 2012a,b,c) and why language can alter on-going emotional experiences and perceptions too (for a review of these findings see Lindquist and Gendron, 2013; Lindquist et al., in press b).

The label-feedback hypothesis suggests that the linguistic and conceptual systems become functionally entwined over the course of development such that activation of concepts in adults tends to activate labels and vice versa (see Lupyan, 2012a). For instance, after learning throughout childhood that scowls occurring in specific contexts (e.g., after an insult) are called “anger,” this knowledge would be brought online to make meaning of future facial movements in situations involving insults. The activation of the label across new situations might further warp visual sensations in a top–down manner, causing scowls made following insults to appear more similar to memories of other scowls made following insults than scowls made when contemplating a colleague’s question. As Lupyan (2012a) points out, modulation of perception by language can be up-regulated when words are explicitly referenced during perception. By contrast, the modulation of perception by language can be down-regulated when the linguistic system is temporarily impaired via verbal interference or other means.

As an example of the up-regulation of language shaping on-going visual perception, a set of studies (Lupyan, 2008; Lupyan and Spivey, 2010a,b) examined the role of verbal labels on participants’ reaction times to identify visual objects. For instance, in one visual identification task, participants were asked to locate a target object (a chair) in an array of non-target objects (tables). At the start of each block, participants were given an example of the stimulus that was the target. On half the trials, before the array of images appeared, participants also received the verbal instructions to “find the category” or “find the chair.” Despite the fact that the word “chair” was redundant with existing instructions, participants were quicker to find the target on these trials, suggesting that labels can help direct attention to certain visual sensations in the environment (Lupyan and Spivey, 2010b).

Importantly, labels appear to modulate sensations in a deep manner by altering which sensations are selected for conscious awareness in the first place. For instance, in one study (Lupyan and Spivey, 2010a) participants completed a task in which they made an object presence vs. absence judgment to briefly presented letters. When participants heard the letter name prior to the judgment, they identified the presence of the letter with greater sensitivity (i.e., judged that it was present when it was in fact present and absent when it was in fact absent). By contrast, a visual cue of the to-be-presented letter did not increase participants’ sensitivity to judge it was present during the trial. In an extension of these findings, participants in a separate study (Lupyan and Ward, 2013) were asked to indicate whether they saw a stimulus presented to one eye during continuous flash suppression (CFS) or not. CFS takes advantage of the binocular nature of vision by directing flashing visual images to one eye and a still image to the other eye. Participants consciously perceive the flashing stimulus because it is dynamic, but the static image is generally suppressed from conscious experience. In Lupyan and Ward’s (2013) study, participants who were presented with valid vs. invalid labels for the object present during CFS actually showed greater sensitivity to detect the presence of a suppressed image.

“Label-feedback” thus explains the myriad ways in which language impacts spatial cognition (Boroditsky, 2001), color perception (Winawer et al., 2007), action perception (Stanfield and Zwaan, 2001; Zwaan et al., 2002) and not least, our own language and emotion findings across adult cognition (for reviews, see Lindquist and Gendron, 2013; Lindquist et al., in press a,b). For instance, in several of our studies, we have down-regulated the label-feedback effect on adult emotion perception by temporarily decreasing participants’ access to the meaning of emotion words via a process called *semantic satiation* (Lindquist et al., 2006; Gendron et al., 2012). In semantic satiation, participants repeat a word out loud 30 times until the meaning of the word becomes temporarily inaccessible. Semantic satiation operates by temporarily disconnecting the phonological form of the word with its meaning (Tian and Huber, 2010). In one of our most recent studies (Gendron et al., 2012), satiating relevant emotion words prior to participants perceiving a face impaired that face’s ability to perceptually prime itself again later in the trial. Perceptual priming is evidenced when seeing a stimulus once causes a person to render faster judgments about the identical stimulus on later presentations and is thought to be mediated by visual processing occurring in the visual cortex of the brain (Grill-Spector, 2008). Specifically, participants repeated a relevant emotion word (e.g., anger) or an irrelevant abstract concept (e.g., idea) out loud 30 times before seeing a facial expression (e.g., Identity 1 depicting a scowl). Later in the trial, participants either saw the same face again (e.g., Identity 1 depicting a scowl) or a face that differed in terms of emotion (e.g., Identity 1 depicting a frown), identity (e.g., Identity 2 depicting a scowl), or both (e.g., Identity 2 depicting a frown). We measured perceptual priming as participants’ speed to render an arbitrary perceptual judgment (i.e. how close or far apart the eyes of the face were) about the second face presented on critical trials when perceptual priming should occur (e.g., when Identity 1 scowls were followed by Identity 1 scowls). We hypothesized that if emotion concepts are routinely involved in emotion perception, then disrupting access to emotion concepts ought to interfere with how an emotional face is perceived, which would in turn impair its ability to perceptually prime itself later in the trial. Consistent with this hypothesis, semantic satiation interfered with the ability of the first face to facilitate judgments made about the subsequently presented face, even though the task involved making an arbitrary perceptual judgment that did not itself require access to emotion concepts. Importantly, our findings were not due to fatigue because satiating an irrelevant word (e.g., “idea”) did not similarly impair a face’s ability to perceptually prime itself later in the trial (Gendron et al., 2012).

Together, these findings suggest that language may not only shape emotion by ultimately helping people acquire knowledge about emotion across development, but language might also more proximally shape emotion by contributing to the ability to make situated conceptualizations of emotion in the moment.

## Implications for the Role of Language in the Acquisition and Use of Emotion

The implications of the role of language in emotion concept acquisition and utilization are vast. In applied arenas, investigations of how infants and children develop emotion knowledge via words could inform interventions for individuals with developmental disorders or maladaptive emotions. Recent evidence suggests that the emotion perception deficits observed in adults with autism are mediated by alexithymic traits (Cook et al., 2013), suggesting an important relationship between autism and emotion concept knowledge. Teaching children to pair their bodily sensations or the facial expressions made by others with words early in life might therefore have a protective effect on children at risk of autism. Such interventions could even be used for infants of alexithymic parents, who are at greater risk of becoming alexithymic themselves and experiencing the associated decrements in health and well-being (Berenbaum and James, 1994; Lumley et al., 1996).

Children with language disorder diagnoses also face emotional difficulties, underscoring the import role of language in emotion. In particular, language impairments seem to impact children’s ability to differentiate between emotions. One study suggested that children with language impairments lack the full range of differentiated positive and negative emotions, and instead simply differentiate their internal states in global terms such as “good” or “bad” (Fujiki et al., 2002). These same children, lacking the emotion differentiation that language enables, also had trouble identifying internal and external cues that would help them regulate their affective states. These findings are ultimately consistent with evidence that learning specific emotion words (e.g., fear, anger, sadness, disgust) helps normally developing children make more differentiated, nuanced situated conceptualizations of other’s affective states. Prior to learning specific emotion words around ages 2–3, normally developing children only seem to understand affective valence (happy vs. sad; Russell and Widen, 2002; Widen and Russell, 2008). Thus, language may help turn a child’s feelings of global “badness” into the differentiated negative emotions that his or her culture’s linguistic structures represent. For example, alexithymic children may be able to perceive and label core affective states in their bodies such as “bad” and “good,” “hurt” and “nice” (which may explain why alexithymic individuals tend to exhibit more somatization disorders: Gulec et al., 2013; Zunhammer et al., 2013; Gulpek et al., 2014; Tominaga et al., 2014), but are unable to differentiate that affect into discrete emotion categories via emotion construction (see Lindquist and Barrett, 2008b for a discussion).

Beyond developing interventions for at-risk children, educational tools encouraging children to label their own and others’ emotions might offer individuals skills that contribute to greater social and emotional well-being. Although young children begin to be able to pair words and emotional expressions across the first several years of life, they might benefit from learning to do this earlier on in childhood. As aforementioned, children who know different discrete emotion words (e.g., “anger” vs. “fear” vs. “sadness”) can correspondingly differentiate between facial expressions of those emotions, and children with parents who label emotions are better at labeling their own emotions even by 36 months of age. Finding practical ways to increase parents’ skill at discussing the bodily, situational, and behavioral aspects of emotions with their children would likely prove beneficial. Classrooms and daycares that routinely ask young children to pair words with facial expressions or to describe the situational and interoceptive features of their feelings may thus produce more emotionally intelligent children who exhibit less worry and depression (Rieffe et al., 2007) and who have superior social and academic outcomes both in the moment and later in life (for reviews see Halberstadt et al., 2001, 2013). Indeed, a recent meta-analysis (Durlak et al., 2011) indicates that children who go through emotion training techniques exhibit an 11-percentile point increase in achievement on grades and standardized test scores and exhibit more prosocial behavior and less emotional distress in daily life.

Language-based emotion interventions might not just apply to children. It is argued that adults with alexithymia have difficulty making situated conceptualizations of their on-going affective states and of others’ facial muscle movements (Lindquist and Barrett, 2008a). If language plays a role in emotion, then one means of treating such individuals would be to have them engage in word-emotion matching tasks. Such tasks could be used in therapists’ offices, in the workplace, or implemented online. We recently found that alexithymic adults have more difficulty matching an emotional face (e.g., an angry face) with another emotional face (e.g., another angry face) than do non-alexithymic adults. Yet alexithymic adults are as quick and sensitive as non-alexithymic individuals when asked to pair a face with a word (Nook et al., in press). These findings suggest that alexithymic individuals may not automatically access words to help make situated conceptualizations of facial expressions, but that when words are provided for them, they can indeed engage in a situated conceptualization of emotion.

The construct of alexithymia has been correlated with a host of psychopathologies (Bagby et al., 1986), and in particular somatization disorders (see Gucht and Heiser, 2003 for review; for a recent review of alexithymia in depression and anxiety, see De Berardis et al., 2008; for a recent review of alexithymia in eating disorders, see Nowakowski et al., 2013; for a recent review and meta-analyses of alexithymia’s connection with schizophrenia, see O’Driscoll et al., 2014; for other recent empirical work on alexithymia’s connection to personality disorders, see Nicolo et al., 2011; Loas et al., 2012); thus, language-based emotion interventions in adults may have clinical relevance to multiple forms of psychopathology. For example, depression and anxiety are both associated with difficulty identifying feelings, while anxiety is specifically associated with difficulty describing feelings (Korkoliakou et al., 2014). Similarly, a recent study on borderline personality disorder (BPD) found that individuals with BPD reacted to empathy inductions with greater personal (rather than empathic) distress and greater difficulty labeling their affective reactions (New et al., 2012). Demiralp et al. (2012) found that individuals with Major Depressive Disorder had less differentiated negative emotion experiences compared with healthy individuals. Our model would suggest that the poor negative emotion differentiation observed in individuals with Major Depressive Disorder may be in part driven by a paucity of conceptual knowledge, which would make it more difficult for individuals to understand and ultimately regulate their negative feelings. Indeed, greater differentiation of one’s emotional states is associated with better emotion regulation (Barrett et al., 2001). Better emotion differentiation may also serve as a protective factor against destructive emotion regulation strategies such as non-suicidal self-injury, as a recent daily diary study of individuals with BPD found (Zaki et al., 2013). Although some research suggests that language (in the form of journaling, for example) can dampen or distance the effects of negative emotions (Pennebaker and Beall, 1986; Wilson and Schooler, 1991; Pennebaker, 1997; Hemenover, 2003), other work suggests that language can also increase the discreteness of an emotion experience, making it easier to regulate (Lieberman et al., 2007; Kassam and Mendes, 2013; Burklund et al., 2014). This literature supports our predictions that when language for emotion concepts is both present and accessible, emotions are constructed as more distinctive and discrete experiences. We suggest that interventions targeted at increasing individuals’ conceptual knowledge and tendency to make situated conceptualizations of emotions (for example, developing a more nuanced understanding of the bodily, behavioral, and situational dimensions of emotions and using that information to help identify which emotion one is feeling) may particularly help individuals who are struggling with high emotional lability and mood dysregulation. We suggest that increased emotion differentiation, supported by improved conceptual and linguistic resources regarding emotion, will increase individuals’ ability to identify and articulate what they are feeling in a way that promotes effective emotion regulation.

Findings suggest that language may also help bilingual or multilingual individuals implicitly regulate their emotions. For instance, it has been argued that because some languages denote differences between emotion categories that others do not (e.g., Vietnamese speakers conceive of shame v. anguish as distinct, whereas English speakers does not; Alvarado and Jameson, 2010), this may promote greater emotion differentiation and thus, greater emotion regulation, when speakers are thinking in this language (for a review see Pavlenko, 2014). Bilingualism might also support emotion regulation by implicitly producing emotional distance when an individual is speaking in their non-dominant language. “Distancing” is an emotion regulation strategy that involves deliberately assuming a detached perspective on the emotional situation (Beck, 1970; Kross and Ayduk, 2011; Kross et al., 2014). A number of studies suggest that multilingual speakers experience less emotional reactivity (measured as skin conductance responses, self-ratings) when presented with words or phrases or when asked to recall events in their non-native language (for a review see Pavlenko, 2014). A second language might therefore implicitly “distance” individuals from the affective value of past and/or present events. However, whether a first or second (or third, etc.) language is likely to serve a distancing function depends on whether that language is a person’s dominant and most frequently used language. In cases in which individuals report that their second language is their dominant and preferred language, those individuals tend to have greater reactivity toward affective words in their second, as compared to their first language (Degner et al., 2012; Simcox et al., 2012).

In addition to important applied implications of a language-emotion link, there are vast theoretical implications for the role of language in emotion. Not least of which, is the implication that emotions are constructed via more basic elements rather than physical types that are only named by words (Barrett, 2006b; Lindquist, 2013; Lindquist et al., in press b). More broadly, the role of language in emotion opens avenues for understanding the cultural relativity of emotions. Previous work on the “taxonomy” of emotions relied on English emotion terms to define the emotions that exist (for example, Shaver et al., 1987; Zinck and Newen, 2007). In reality, we believe that these types of measures catalog conceptual knowledge about emotion categories, and in most cases, English emotion conceptual knowledge in particular. However, as Wierzbicka and others have argued, mapping human experience solely based on English language terms fails to understand the highly variable nature of emotion across cultures (Wierzbicka, 2009).

The CAT recognizes the power of language in emotion and instead predicts that the precise emotions experienced by a given person in a given culture will depend on the emotion concepts available to that person. Instead of hypothesizing that basic emotion categories are given by specific structures in the brain, the CAT envisions emotion terms such as “happiness,” “sadness,” “fear,” “disgust,” and “anger” as abstract concepts that become “essence placeholders” for distributions of conceptual knowledge about embodied mental states. Such conceptual knowledge feeds forward in a given instance of experience to help predict the meaning of bodily sensations in a given context. The brain infers that the current subjective state most closely matches a certain population of instances characterized as, e.g., “sadness” (in English) or perhaps an entirely different population of instances in another language. Critically, since different languages provide different morphological placeholders for distributions of embodied knowledge, this could cause individuals to segment and experience their momentary bodily states in either subtly different or even quite distinct ways. Recognizing the role of language in emotion may thus help scientists better measure and document the individual differences and cultural relativity underlying emotion categories in a way that can plot new directions for the study of emotion. We look forward to future directions in language-emotion research that will assess the ways in which language acquisition and utilization shape how humans experience the emotional world across the lifespan.

## Conflict of Interest Statement

The authors declare that the research was conducted in the absence of any commercial or financial relationships that could be construed as a potential conflict of interest.

## Footnotes

1 Importantly, situated conceptualization does not happen because people *consciously* categorize ambiguous feelings or situations. It is an effortless and not necessarily conscious mechanism of how the human brain works, as it masters and makes meaning of the information and statistical regularities of experience. The analogy is that the brain uses knowledge from prior experience to transform wavelengths of visible light into the perception of a specific color (Barrett, 2006b). This process differs based on the lighting present in a room and even the other colors present in the context (see Bruner et al., 1951).

2 The natural kind view was prevalent for the latter half of the 20th century, but recent evidence from behavior, peripheral physiology, and neuroscience has amassed to suggest that emotions such as anger, disgust, fear, happiness, sadness, etc. are not physical types with consistent and specific behavioral (Barrett, 2006b; Mauss and Robinson, 2009) and physiological outputs (Cacioppo et al., 2000; Barrett, 2006b; Mauss and Robinson, 2009; Quigley and Barrett, 2014) that derive from specific circuits or regions in the brain (Kober et al., 2008; Lindquist et al., in press a; Kassam et al., 2013; Touroutoglou et al., in press).

3 Of note, the CAT exclusively makes predictions for words that name specific emotion categories in a given language (e.g., “anger,” “disgust,” “fear,” in English and other culturally relevant terms in other languages). We are not referring to words that name other categories (e.g., “mother,” “murder”) that might themselves have emotional connotations. These words, if they help construct emotions, likely do so via less proximal mechanisms. For instance, the word “mother” might prime the word “love,” which in turn might cause an individual to access the relevant body states, exteroceptive sensations, and conceptual knowledge associated with that emotion concept (for a discussion of the embodiment of emotion words see Barrett and Lindquist, 2008). Accessing or “simulating” the relevant affective and sensorimotor concomitants of the category “love” could in turn cause a person to start to feel an instance of love toward her mother. However, this process is clearly different from the process of emotion construction we are proposing in which the conceptual knowledge associated with the word “love” is being used in the moment make a situated conceptualization of the pleasant affect that is experienced when talking to one’s mother on the phone, when hugging one’s mother, etc. For research on the emotional connotations of words and implications for psycholinguistics, we point interested readers to Altarriba et al. (1999), Kousta et al. (2011) and Borghi and Binkofski (2014). We also point interested readers to fascinating research demonstrating that the emotional connotations of words in second languages are not as intense as the emotional connotations of words in first languages (for review see Harris et al., 2006; Opitz and Degner, 2012).

4 Rational constructivism is of the broader class of psychological constructionist views of the mind (Barrett, 2009; Lindquist and Barrett, 2012; Barrett and Satpute, 2013).

5 Although for evidence that 15-month old infants can use music as a cue for acquiring categories, see Roberts and Jacob (1991).

6 Evidence claiming that infants can reliably differentiate between different facial expression on the basis of something other than valence may be driven by differences in the perceptual regularities present in stimuli that are not related to the emotion category itself (e.g., the presence of teeth; Caron et al., 1985).

## References

[B1] Allport F. H. (1924). *Social Psychology*. New York: Houghton Miﬄin.

[B2] Altarriba J. Bauer L. M. Benvenuto C. (1999). Concreteness, context-availability, and imageability ratings and word associations for abstract, concrete, and emotion words. *Behav. Res. Methods Instrum. Comput.* 31 578–602 10.3758/BF03200738 10633977

[B3] Alvarado N. Jameson K. (2010). Shared knowledge about emotion among Vietnamese and English bilingual and monolingual speakers. *J. Cross Cult. Psychol.* 42 963–982 10.1177/0022022110381125

[B4] Aslin R. N. Newport E. L. (2012). Statistical learning: from acquiring specific items to forming general rules. *Curr. Dir. Psychol. Sci.* 21 170–176 10.1177/0963721412436806 24000273 PMC3758750

[B5] Aslin R. N. Saffran J. R. Newport E. L. (1998). Computation of conditional probability statistics by 8-month-old infants. *Psychol. Sci.* 9 321–324 10.1111/1467-9280.00063

[B6] Bagby R. M. Taylor G. J. Ryan D. (1986). Toronto Alexithymia Scale: relationship with personality and psychopathology measures. *Psychother. Psychosomat.* 45 207–215 10.1159/000287950 3588819

[B7] Bar M. (2007). The proactive brain: using analogies and associations to generate predictions. *Trends Cogn. Sci.* 11 280–289 10.1016/j.tics.2007.05.005 17548232

[B8] Barrera M. E. Maurer D. (1981). The perception of facial expressions by the three-month-old. *Child Dev.* 52 203–206 10.2307/1129231 7238144

[B9] Barrett L. F. (2006a). Are emotions natural kinds? *Perspect. Psychol. Sci.* 1 28–58 10.1111/j.1745-6916.2006.00003.x 26151184

[B10] Barrett L. F. (2006b). Solving the emotion paradox: categorization and the experience of emotion. *Pers. Soc. Psychol. Rev.* 10 20–46 10.1207/s15327957pspr1001_2 16430327

[B11] Barrett L. F. (2009). The future of psychology: connecting mind to brain. *Perspect. Psychol. Sci.* 4 326–339 10.1111/j.1745-6924.2009.01134.x 19844601 PMC2763392

[B12] Barrett L. F. (2012). Emotions are real. *Emotion* 12 413–429 10.1037/a0027555 22642358

[B13] Barrett L. F. (2013). Psychological construction: a darwinian approach to the science of emotion. *Emot. Rev.* 5 379–389 10.1177/1754073913489753

[B14] Barrett L. F. (2014). The conceptual act theory: a précis. *Emot. Rev.* 6 292–297 10.1177/1754073914534479

[B15] Barrett L. F. Bliss-Moreau E. (2009). Affect as a psychological primitive. *Adv. Exp. Soc. Psychol.* 41 167–218 10.1016/S0065-2601(08)00404-8 20552040 PMC2884406

[B16] Barrett L. F. Lindquist K. A. (2008). “The embodiment of emotion,” in *Embodied Grounding: Social, Cognitive, Affective, and Neuroscientific Approaches*, eds Semin G. R. Smith G. R. (New York: Cambridge University Press).

[B17] Barrett L. F. Lindquist K. A. Gendron M. (2007a). Language as context for the perception of emotion. *Trends Cogn. Sci.* 11 327–332 10.1016/j.tics.2007.06.003 17625952 PMC2225544

[B18] Barrett L. F. Ochsner K. N. Gross J. J. (2007b). “On the automaticity of emotion,” in *Social Psychology and the Unconscious: The Automaticity of Higher Mental Processes*, ed.Bargh J. (New York: Psychology Press), 173–217.

[B19] Barrett L. F. Gross J. Conner T. Benvenuto M. (2001). Knowing what you’re feeling and knowing what to do about it: mapping the relation between emotion differentiation and emotion regulation. *Cogn. Emot.* 15 713–724 10.1080/02699930143000239

[B20] Barrett L. F. Satpute A. B. (2013). Large-scale brain networks in affective and social neuroscience: towards an integrative functional architecture of the brain. *Curr. Opin. Neurobiol.* 23 361–372 10.1016/j.conb.2012.12.012 23352202 PMC4119963

[B21] Barsalou L. W. (1999). Perceptual symbol systems. *Behav. Brain Sci.* 22 577–609 10.1017/S0140525X99002149 11301525

[B22] Barsalou L. W. (2009). Simulation, situated conceptualization, and prediction. *Philos. Trans. R. Soc. Lond. Biol. Sci.* 364 1281–1289 10.1098/rstb.2008.0319 19528009 PMC2666716

[B23] Barsalou L. W. (2012). “The human conceptual system,” in *The Cambridge Handbook of Psycholinguistics,* eds Spivey M. McRae K. Joanisse M. Spivey M. McRae K. Joanisse M. (New York: Cambridge University Press), 239–258 10.1017/CBO9781139029377.017

[B24] Barsalou L. W. Santos A. Simmons W. K. Wilson C. D. (2008). “Language and simulation in conceptual processing,” in *Symbols, Embodiment, Meaning,* eds De Vega M. Glenberg A. M. Graesser A. C. (Oxford: Oxford Univerity Press), 245–283.

[B25] Barsalou L. W. Wiemer-Hastings K. (2005). “Situating abstract concepts,” in *Grounding Cognition: The Role of Perception and Action in Memory, Language, and Thought*, ed.Pecher D. (New York: Cambridge University Press), 129–163 10.1017/CBO9780511499968.007

[B26] Beck A. T. (1970). Cognitive therapy: nature and relation to behavior therapy. *Behav. Ther.* 1 184–200 10.1016/S0005-7894(70)80030-2 27993332

[B27] Behl-Chadha G. (1996). Basic-level and superordinate-like categorical representations in early infancy. *Cognition* 60 105–141 10.1016/0010-0277(96)00706-8 8811742

[B28] Berenbaum H. James T. (1994). Correlates and retrospectively reported antecedents of alexithymia. *Psychosom. Med.* 56 345–352 10.1097/00006842-199407000-00011 7972618

[B29] Bergelson E. Swingley D. (2012). At 6-9 months, human infants know the meanings of many common nouns. *Proc. Natl. Acad. Sci. U.S.A.* 109 3253–3258 10.1073/pnas.1113380109 22331874 PMC3295309

[B30] Borghi A. M. Binkofski F. (2014). *Words As social Tools: An Embodied View on Abstract Concepts. SpringerBriefs in Cognition Series*. New York: Springer 10.1007/978-1-4614-9539-0

[B31] Boroditsky L. (2001). Does language shape thought? Mandarin and English speakers’ conceptions of time. *Cogn. Psychol.* 43 1–22 10.1006/cogp.2001.0748 11487292

[B32] Boroditsky L. (2011). How language shapes thought. *Sci. Am.* 304 62–65. 21319543 10.1038/scientificamerican0211-62

[B33] Bowerman M. (1988). “The ’no negative evidence’ problem: how do children avoid constructing an overly general grammar?” in *Explaining Language Universals*, ed.Hawkins J. A. (Oxford: Basil Blackwell), 73–101.

[B34] Bruner J. S. Postman L. Rodrigues J. (1951). Expectation and the perception of color. *Am. J. Psychol.* 64 216–227 10.2307/1418668 14829628

[B35] Burklund L. J. Creswell J. D. Irwin M. Lieberman M. D. (2014). The common and distinct neural bases of affect labeling and reappraisal in healthy adults. *Front. Psychol.* 5:221 10.3389/fpsyg.2014.00221 PMC3970015 24715880

[B36] Cabanac M. (2002). What is emotion? *Behav. Process.* 60 69–83 10.1016/S0376-6357(02)00078-5 12426062

[B37] Cacioppo J. T. Berntson C. G. Larsen J. T. Poehlmann K. M. Ito T. A. (2000). “The psychophysiology of emotion,” in *Handbook of Emotions*, eds Lewis M. J. Jones M. H. (New York: Guilford), 173–191.

[B38] Campos J. J. Bertenthal B. I. Kermoian R. (1992). Early experience and emotional development: the emergence of wariness of heights. *Psychol. Sci.* 3 61–64 10.1111/j.1467-9280.1992.tb00259.x

[B39] Cannon W. B. (1921). The James-Lange theory of emotions: a critical examination and an alternative theory. *Am. J. Psychol.* 38 106–124 10.2307/1415404 3322057

[B40] Carey S. (2011). Précis of “The Origin of Concepts.” *Behav. Brain Sci.* 34 113–124 10.1017/S0140525X10000919 21676291 PMC3489495

[B41] Caron R. F. Caron A. J. Myers R. S. (1985). Do infants see emotional expressions in static faces? *Child Dev.* 56 1552–1560 10.2307/1130474 4075873

[B42] Castro V. L. Halberstadt A. G. Lozada F. T. Craig A. B. (2014). Parents’ emotion-related beliefs, behaviours, and skills predict children’s recognition of emotion. *Infant Child Dev.* 24 1–22 10.1002/icd.1868 PMC4437215 26005393

[B43] Cervantes C. Callanan M. A. (1998). Labels and explanations in mother-child emotion talk: age and gender differentiation. *Dev. Psychol.* 34 88–98 10.1037/0012-1649.34.1.88 9471007

[B44] Clark A. (2013). Whatever next? Predictive brains, situated agents, and the future of cognitive science. *Behav. Brain Sci.* 36 181–204 10.1017/S0140525X12000477 23663408

[B45] Clore G. L. Ortony A. (1991). What more is there to emotion concepts than prototypes? *J. Pers. Soc. Psychol.* 60 48–50 10.1037//0022-3514.60.1.48

[B46] Clore G. L. Ortony A. (2008). “Appraisal theories: how cognition shapes affect into emotion,” in *Handbook of Emotions*, eds Lewis M. Haviland-Jones J. M. Barret L. F. (New York: Guilford Press), 628–642.

[B47] Clore G. L. Ortony A. (2013). Psychological construction in the OCC model of emotion. *Emot. Rev.* 5 335–343 10.1177/1754073913489751 25431620 PMC4243519

[B48] Cook R. Brewer R. Shah P. Bird G. (2013). Alexithymia, not autism, predicts poor recognition of emotional facial expressions. *Psychol. Sci.* 24 723–732 10.1177/0956797612463582 23528789

[B49] Cunningham W. A. Dunfield K. A. Stillman P. E. (2013). Emotional states from affective dynamics. *Emot. Rev.* 5 344–355 10.1177/1754073913489749

[B50] De Berardis D. Campanella D. Serroni N. Sepede G. Carano A. Conti C. (2008). The impact of alexithymia on anxiety disorders: a review of the literature. *Curr. Psychiatry Rev.* 4 80–86 10.2174/157340008784529287

[B51] Degner J. Doycheva C. Wentura D. (2012). It matters how much you talk: on the automaticity of affective communication of first and second language words. *Biling. Lang. Cogn.* 15 181–189 10.1017/S1366728911000095

[B52] Demiralp E. Thompson R. J. Mata J. Barrett L. F. Ellsworth P. C. Demiralp M. (2012). Feeling blue or turquoise? Emotional differentiation in major depressive disorder. *Psychol. Sci.* 23 1410–1416 10.1177/0956797612444903 23070307 PMC4004625

[B53] Dewar K. Xu F. (2009). Do early nouns refer to kinds or distinct shapes? Evidence from 10-month-old infants. *Psychol. Sci.* 20 252–257 10.1111/j.1467-9280.2009.02278.x 19175526

[B54] Dunn J. Bretherton I. Munn P. (1987). Conversations about feeling states between mothers and their young children. *Dev. Psychol.* 23 132–139 10.1037/0012-1649.23.1.132

[B55] Dunn J. Brown J. Slomkowski C. Tesla C. Youngblade L. (1991). Young children’s understanding of other people’s feelings and beliefs: individual differences and their antecedents. *Child Dev.* 62 1352–1366 10.2307/1130811 1786720

[B56] Dunsmore J. C. Halberstadt A. G. (1997). “How does family emotional expressiveness affect children’s schemas?” in *The Communication of Emotion: Current Research From Diverse Perspectives*, ed.Barrett K. C. (San Francisco, CA: Jossey-Bass), 45–68 10.1002/cd.23219977704 9457805

[B57] Dunsmore J. C. Her P. Halberstadt A. G. Perez-Rivera M. B. (2009). Parents’ beliefs about emotions and children’s recognition of parents’ emotions. *J. Nonverbal Behav.* 33 121–140 10.1007/s10919-008-0066-6 20160992 PMC2755301

[B58] Durlak J. A. Weissberg R. P. Dymnicki A. B. Taylor R. D. Schellinger K. B. (2011). The impact of enhancing students’ social and emotional learning: a meta-analysis of school-based universal interventions. *Child Dev.* 82 405–432 10.1111/j.1467-8624.2010.01564.x 21291449

[B59] Eimas P. D. Miller J. L. (1992). Organization in the perception of speech by young infants. *Psychol. Sci.* 3 340–345 10.1111/j.1467-9280.1992.tb00043.x

[B60] Ekman P. Cordaro D. (2011). What is meant by calling emotions basic. *Emot. Rev.* 3 364–370 10.1177/1754073911410740

[B61] Ekman P. Oster H. (1979). Facial expressions of emotion. *Annu. Rev. Psychol.* 30 527–554 10.1146/annurev.ps.30.020179.002523

[B62] Fontaine J. R. J. Scherer K. R. Soriano C. (2013). “A paradigm for a multidisciplinary investigation of the meaning of emotion terms,” in *Components of Emotional Meaning: A Sourcebook*, eds Fontaine J. R. J. Scherer K. R. Soriano C. (Oxford: Oxford University Press). 10.1093/acprof:oso/9780199592746.001.0001

[B63] Fugate J. Gouzoules H. Barrett L. F. (2010). Reading chimpanzee faces: evidence for the role of verbal labels in categorical perception of emotion. *Emotion* 10 544–554 10.1037/a0019017 20677871 PMC2949420

[B64] Fujiki M. Brinton B. Clarke D. (2002). Emotion regulation in children with specific langauge impairment. *Lang. Speech Hear. Ser. Sch.* 33 102–111 10.1044/0161-1461(2002/008) 27764463

[B65] Fukunishi I. Paris W. (2001). Intergenerational association of alexithymic characteristics for college students and their mothers. *Psychol. Rep.* 89 77–84 10.2466/pr0.2001.89.1.77 11729556

[B66] Gebhart A. L. Newport E. L. Aslin R. N. (2009). Statistical learning of adjacent and non-adjacent dependencies among non-linguistic sounds. *Psychonom. Bull. Rev.* 16 486–490 10.3758/PBR.16.3.486 PMC2799498 19451373

[B67] Gelman S. A. Markman E. M. (1987). Young children’s inductions from natural kinds: the role of categories and appearances. *Child Dev.* 58 1532–1541 10.2307/1130693 3691200

[B68] Gendron M. Barrett L. F. (2009). Reconstructing the past: a century of ideas about emotion in psychology. *Emot. Rev.* 1 316–339 10.1177/1754073909338877 20221412 PMC2835158

[B69] Gendron M. Lindquist K. A. Barsalou L. W. Barrett L. F. (2012). Emotion words shape emotion percepts. *Emotion* 12 314–325 10.1037/a0026007 22309717 PMC4445832

[B70] Gendron M. Roberson D. van der Vyver J. M. Barrett L. F. (2014). Perceptions of emotion from facial expressions are not culturally universal: evidence from a remote culture. *Emotion* 14 251–262 10.1037/a0036052 24708506 PMC4752367

[B71] Gerhardt K. J. Abrams R. M. (2000). Fetal exposures to sound and vibroacoustic stimulation. *J. Perinatol.* 20 S20–S29 10.1038/sj.jp.7200446 11190697

[B72] Glenberg A. M. Gallese V. (2012). Action-based language: a theory of language acquisition, comprehension, and production. *Cortex* 48 905–922 10.1016/j.cortex.2011.04.010 21601842

[B73] Goldstone R. (1994). Influences of categorization on perceptual discrimination. *J. Exp. Psychol.* 2 178–200 10.1037/0096-3445.123.2.178 8014612

[B74] Gottman J. M. Katz L. F. Hooven C. (1996). Parental meta-emotion philosophy and the emotional life of families: theoretical models and preliminary data. *J. Fam. Psychol.* 10 243–268 10.1037//0893-3200.10.3.243

[B75] Grill-Spector K. (2008). “Visual priming,” in *Leaning and Memory: A Comprehensice Reference*, eds Eichenbaum H. Bryne J. (Oxford: Elsevier), 219–236 10.1016/B978-012370509-9.00130-3

[B76] Gucht V. D. Heiser W. (2003). Alexithymia and somatisation: a quantitative review of the literature. *J. Psychosom. Res.* 54 425–434 10.1016/S0022-3999(02)00467-1 12726898

[B77] Gulec M. Y. Altintas M. Inanc L. Bezgin C. H. Koca E. K. Gulec H. (2013). Effects of childhood trauma on somatization in major depressive disorder: the role of alexithymia. *J. Affect. Disord.* 146 137–141 10.1016/j.jad.2012.06.033 22884234

[B78] Gulpek D. Kelemence K. F. Kesebir S. Bora O. (2014). Alexithymia in patients with conversion disorder. *Nord. J. Psychiatry* 68 300–305 10.3109/08039488.2013.814711 23902128

[B79] Hakim-Larson J. Parker A. Lee C. Goodwin J. Voelker S. (2006). Measuring parental meta-emotion: psychometric properties of the emotion-related parenting styles self-test. *Early Educ. Dev.* 17 229–251 10.1207/s15566935eed1702_2

[B80] Halberstadt A. G. Denham S. A. Dunsmore J. C. (2001). Affective social competence. *Soc. Dev.* 10 79–119 10.1111/1467-9507.00150

[B81] Halberstadt A. G. Lozada F. T. (2011). Emotional development in infancy through the lens of culture. *Emot. Rev.* 3 158–168 10.1177/1754073910387946

[B82] Halberstadt A. G. Parker A. E. Castro V. L. (2013). “Nonverbal communication: development perspectives,” in *Handbook of Communication Science*, eds Hall J. A. Knapp M. L. (Berlin: Springer).

[B83] Harris C. L. Gleason J. B. Aycicegi A. (2006). “When is a first language more emotional? Psychophysiological evidence from bilingual speakers,” in *Bilingual Minds: Emotional Experience, Expression, and Representation*, ed.Pavlenko A. (Clevedon: Mulilingual Matters).

[B84] Harris P. de Rosnay M. Pons F. (2005). Language and children’s understanding of mental states. *Curr. Dir. Psychol. Sci.* 14 69–73 10.1111/j.0963-7214.2005.00337.x

[B85] Hemenover S. H. (2003). The good, the bad, and the healthy: impacts of emotional disclosure of trauma on resilient self-concept and psychological distress. *Pers. Soc. Psychol. Bull.* 29 1236–1244 10.1177/0146167203255228 15189585

[B86] Hepper P. G. Shahidullah B. S. (1994). Development of fetal hearing. *Arch. Dis. Child.* 71 F81–F87 10.1136/fn.71.2.F81 7979483 PMC1061088

[B87] Hunt W. A. (1941). Recent developments in the field of emotion. *Psychol. Bull.* 38 249–276 10.1037/h0054615

[B88] Izard C. E. (1971). *The Face of Emotion,* Vol. 23 New York: Appleton-Century-Crofts.

[B89] Izard C. E. (1978). On the ontogenesis of emotions and emotion-cognition relationships in infancy. *Dev. Affect.* 1 389–413 10.1007/978-1-4684-2616-8_17

[B90] Izard C. E. (2007). Basic emotions, natural kinds, emotion schemas, and a new paradigm. *Perspect. Psychol. Sci.* 2 260–280 10.1111/j.1745-6916.2007.00044.x 26151969

[B91] Izard C. E. (2011). Forms and functions of emotions: matters of emotion-cognition interactions. *Emot. Rev.* 3 371–378 10.1177/1754073911410737

[B92] Kassam K. S. Markey A. R. Cherkassky V. L. Loewenstein G. Just M. A. (2013). Identifying emotions on the basis of neural activation. *PLoS ONE* 8:e66032 10.1371/journal.pone.0066032 PMC3686858 23840392

[B93] Kassam K. S. Mendes W. B. (2013). The effects of measuring emotion: physiological reactions to emotional situations depend on whether someone is asking. *PLoS ONE* 8:e64959 10.1371/journal.pone.0064959PONE-D-12-38157 PMC3680163 23785407

[B94] Kiefer M. Barsalou L. W. (2013). “Grounding the human conceptual system in perception, action, and internal states,” in *Action Science: Foundations of an Emerging Discipline*, eds Prinz W. Beisert M. Herwig A. (Cambridge, MA: MIT Press), 381–407 10.7551/mitpress/9780262018555.003.0015

[B95] Kober H. Barrett L. F. Joseph J. Bliss-Moreau E. Lindquist K. A. Wager T. D. (2008). Functional grouping and cortical-subcortical interactions in emotion: a meta-analysis of neuroimaging studies. *Neuroimage* 42 998–1031 10.1016/j.neuroimage.2008.03.059 18579414 PMC2752702

[B96] Korkoliakou P. Christodoulou C. Kouris A. Porichi E. Efstathiou V. Kaloudi F. (2014). Alexithymia, anxiety and depression in patients with psoriasis: a case-control study. *Ann. Gen. Psychiatry* 13 38–43 10.1186/s12991-014-0038-37 25520742 PMC4269099

[B97] Kousta S. Vigliocco G. Vinson D. P. Andrews M. Del Campo E. (2011). The representation of abstract words: why emotion matters. *J. Exp. Psychol. Gen.* 140 14–34 10.1037/a0021446 21171803

[B98] Kreibig S. D. (2010). Autonomic nervous system activity in emotion: a review. *Biol. Psychol.* 84 394–421 10.1016/j.biopsycho.2010.03.010 20371374

[B99] Krogh L. Vlach H. A. Johnson S. P. (2013). Statistical learning across development: flexible yet constrained. *Front. Lang. Sci.* 3:598 10.3389/fpsyg.2012.00598 PMC3576810 23430452

[B100] Kross E. Ayduk O. (2011). Making meaning out of negative experiences by self-distancing. *Curr. Dir. Psychol. Sci.* 20 187–191 10.1177/0963721411408883

[B101] Kross E. Bruehlman-Senecal E. Park J. Burson A. Dougherty A. Shablack H. (2014). Self-talk as a regulatory mechanism: how you do it matters. *J. Pers. Soc. Psychol.* 106 304–324 10.1037/a0035173 24467424

[B102] Kuhl P. K. (2004). Early language acquisition: cracking the speech code. *Nat. Rev. Neurosci.* 5 831–843 10.1038/nrn1533 15496861

[B103] Lewis M. (2000). “The emergence of human emotions,” in *Handbook of Emotions*, eds Lewis M. Haviland-Jones J. M. (New York: Guilford), 265–280.

[B104] Lieberman M. D. Eisenberger N. I. Crockett M. J. Tom S. M. Pfeifer J. H. Way B. M. (2007). Putting feelings into words. *Psychol. Sci.* 18 421–428 10.1111/j.1467-9280.2007.01916.x 17576282

[B105] Lindquist K. A. (2013). Emotions emerge from more basic psychological ingredients: a modern psychological constructionist model. *Emot. Rev.* 5 356–368 10.1177/1754073913489750

[B106] Lindquist K. A. Barrett L. F. (2008a). Constructing emotion: the experience of fear as a conceptual act. *Psychol. Sci.* 19 898–903 10.1111/j.1467-9280.2008.02174.x 18947355 PMC2758776

[B107] Lindquist K. A. Barrett L. F. (2008b). “Emotional complexity,” in *Handbook of Emotions,* 3rd Edn, eds Lewis M. Haviland-Jones J. M. Barrett L. F. (New York: Guilford).

[B108] Lindquist K. A. Barrett L. F. (2012). A functional architecture of the human brain: insights from emotion. *Trends Cogn. Sci.* 16 533–554 10.1016/j.tics.2012.09.005 23036719 PMC3482298

[B109] Lindquist K. A. Barrett L. F. Bliss-Moreau E. Russell J. A. (2006). Language and the perception of emotion. *Emotion* 6 125–138 10.1037/1528-3542.6.1.125 16637756

[B110] Lindquist K. A. Gendron M. (2013). What’s in a word: language constructs emotion perception. *Emot. Rev.* 5 66–71 10.1177/1754073912451351

[B111] Lindquist K. A. Gendron M. Barrett L. F. Dickerson B. C. (2014). Emotion, but not affect perception, is impaired with semantic memory loss. *Emotion* 14 375–387 10.1037/a0035293 24512242 PMC4119962

[B112] Lindquist K. A. Gendron M. Satpute A. (in press a). “Language and emotion: putting feelings into words and words into feelings,” in *Handbook of Emotions,* 4th Edn, eds Lewis M. Haviland-Jones J. Barrett L. F. (New York: Guildford).

[B113] Lindquist K. A. Satpute A. Gendron M. (in press b). Does language domore than communicate emotion? *Curr. Dir. Psychol. Sci.* 10.1177/0963721414553440 PMC4428906 25983400

[B114] Lindquist K. A. Wager T. D. Kober H. Bliss-Moreau E. Barrett L. F. (2012). The brain basis of emotion: a meta-analytic review. *Behav. Brain Sci.* 35 121–143 10.1017/S0140525X11000446 22617651 PMC4329228

[B115] Loas G. Speranza M. Pham-Scottez A. Perez-Diaz F. Corcos M. (2012). Alexithymia in adolescents with borderline personality disorder. *J. Psychosom. Res.* 72 147–152 10.1016/j.jpsychores.2011.11.006 22281457

[B116] Lumley M. A. Mader C. Gramzow J. Papineau K. (1996). Family factors related to alexithymia characteristics. *Psychosom. Med.* 58 211–216 10.1097/00006842-199605000-00003 8771619

[B117] Lupyan G. (2008). The conceptual grouping effect: categories matter (and named categories matter more). *Cognition* 108 566–577 10.1016/j.cognition.2008.03.009 18448087

[B118] Lupyan G. (2012a). Linguistically modulated perception and cognition: the label-feedback hypothesis. *Front. Psychol.* 3:54 10.3389/fpsyg.2012.00054 PMC3297074 22408629

[B119] Lupyan G. (2012b). “What do words do? Toward a theory of language-augmented thought,” in *Psychology of Learning and Motivation*, Vol. 57 ed.Ross B. H. (Oxford: Elsevier), 255–297.

[B120] Lupyan G. (2012c). Language augmented prediction. *Front. Psychol.* 3:422 10.3389/fpsyg.2012.00422 PMC3483055 23112777

[B121] Lupyan G. Rakison D. H. McClelland J. L. (2007). Language is not just for talking: redundant labels facilitate learning of novel categories. *Psychol. Sci.* 18 1077–1083 10.1111/j.1467-9280.2007.02028.x 18031415

[B122] Lupyan G. Spivey M. J. (2010a). Making the invisible visible: verbal but not visual cues enhance visual detection. *PLoS ONE* 5:e11452 10.1371/journal.pone.0011452 PMC2898810 20628646

[B123] Lupyan G. Spivey M. J. (2010b). Redundant spoken labels facilitate perception of multiple items. *Attent. Percept. Psychophys.* 72 2236–2253 10.3758/APP 21097866

[B124] Lupyan G. Ward E. J. (2013). Language can boost otherwise unseen objects into visual awareness. *Proc. Natl. Acad. Sci. U.S.A.* 110 14196–14201 10.1073/pnas.1303312110 23940323 PMC3761589

[B125] Mauss I. B. Robinson M. D. (2009). Measures of emotion: a review. *Cogn. Emot.* 23 209–237 10.1080/02699930802204677 19809584 PMC2756702

[B126] Maye J. Werker J. F. Gerken L. (2002). Infant sensitivity to distributional information can affect phonetic discrimination. *Cognition* 82 B101–B111 10.1016/S0010-0277(01)00157-3 11747867

[B127] Meteyard L. Cuadrado S. R. Bahrami B. Vigliocco G. (2012). Coming of age: a review of embodiment and the neuroscience of semantics. *Cortex* 48 788–804 10.1016/j.cortex.2010.11.002 21163473

[B128] Moon C. Lagercrantz H. Kuhl P. K. (2013). Language experienced in utero affects vowel perception after birth: a two-country study. *Acta Paediatr.* 102 156–160 10.1111/apa.12098 23173548 PMC3543479

[B129] New A. S. Rot M. Ripoll L. H. Perez-Rodriguez M. M. Lazarus S. Zipursky E. (2012). Empathy and alexithymia in borderline personality disorder: clinical and laboratory measures. *J. Personal. Disord.* 26 660–675 10.1521/pedi.2012.26.5.660 23013336

[B130] Nicolo G. Semerari A. Lysaker P. H. Dimaggio G. Conti L. D’Angerio S. (2011). Alexithymia in personality disorders: correlations with symptoms and interpersonal functioning. *Psychiatry Res.* 190 37–42 10.1016/j.psychres.2010.07.046 20800288

[B131] Nook E. Lindquist K. A. Zaki J. (in press). The construction of emotion in faces: words as context. *Emotion*.

[B132] Nowakowski M. E. McFarlane T. Cassin S. (2013). Alexithymia and eating disorders: a critical review of the literature. *J. Eat. Disord.* 1 21 10.1186/2050-2974-1-21 PMC4081716 24999402

[B133] Oaksford M. Chater N. (2009). Précis of bayesian rationality: the probabilistic approach to human reasoning. *Behav. Brain Sci.* 32 69–84 10.1017/S0140525X09000284 19210833

[B134] O’Driscoll C. Laing J. Mason O. (2014). Cognitive emotion regulation strategies, alexithymia, and dissociation in schizophrenia: a review and meta-analysis. *Clin. Psychol. Rev.* 34 482–495 10.1016/j.cpr.2014.07.002 25105273

[B135] Oosterwijk S. Lindquist K. A. Anderson E. C. Dautoff R. Moriguchi Y. Barrett L. F. (2012). States of mind: emotions, body feelings, and thoughts share distributed neural networks. *Neuroimage* 63 2110–2128 10.1016/j.neuroimage.2012.05.079 22677148 PMC3453527

[B136] Oosterwijk S. Touroutoglou A. Lindquist K. A. (2014). “The neuroscience of construction: what neuroimaging can tell us about how the brain creates the mind,” in *The Psychological Construction of Emotion*, eds Barrett L. G. Russell J. A. (New York: Guilford).

[B137] Opitz B. Degner J. (2012). Emotionality in a second language: it’s a matter of time. *Neuropsychologia* 50 1961–1196 10.1016/j.neuropsychologia.2012.04.021 22569217

[B138] Partanen E. Kujala T. Näätänen R. Liitola A. Sambeth A. Huotilainen M. (2013). Learning-induced neural plasticity of speech processing before birth. *Proc. Natl. Acad. Sci. U.S.A.* 110 15145–15150 10.1073/pnas.1302159110 23980148 PMC3773755

[B139] Panksepp J. (2011). Empathy and the laws of affect. *Science* 334 1358–1359 10.1126/science.1216480 22158811

[B140] Pavlenko A. (2014). *The Bilingual Mind: And What it Tells us About Language and Thought*. New York: Cambridge University Press 10.1017/CBO9781139021456

[B141] Pennebaker J. W. (1997). Writing about emotional experiences as a therapeutic process. *Psychol. Sci.* 8 162–166 10.1111/j.1467-9280.1997.tb00403.x

[B142] Pennebaker J. W. Beall S. (1986). Confronting a traumatic event: toward an understanding of inhibition and disease. *J. Abnorm. Psychol.* 95 274–281 10.1037/0021-843X.95.3.274 3745650

[B143] Plunkett K. Hu J.-F. Cohen L. B. (2008). Labels can override perceptual categories in early infancy. *Cognition* 106 665–681 10.1016/j.cognition.2007.04.003 17512515

[B144] Quigley K. S. Barrett L. F. (2014). Is there consistency and specificity of autonomic changes during emotional episodes? Guidance from the conceptual act theory and psychophysiology. *Biol. Psychol.* 98 82–94 10.1016/j.biopsycho.2013.12.013 24388802 PMC4041070

[B145] Quinn P. C. Eimas P. D. (1996). Perceptual cues that permit categorical differentiation of animal species by infants. *J. Exp. Child Psychol.* 211 189–211 10.1006/jecp.1996.0047 8812045

[B146] Rieffe C. Meerum Terwogt M. Petrides K. V. Cowan R. Miers A. C. Tolland A. (2007). Psychometric properties of the emotion awareness questionnaire for children. *Pers. Individ. Dif.* 43 95–105 10.1016/j.paid.2006.11.015

[B147] Rivera-Gaxiola M. Silva-Pereyra J. Kuhl P. K. (2005). Brain potentials to native and non-native speech contrasts in 7- and 11-month-old American infants. *Dev. Sci.* 8 162–172 10.1111/j.1467-7687.2005.00403.x 15720374

[B148] Roberts K. Jacob M. (1991). Linguistic versus attentional influences on nonlinguistic categorization in 15-month-old infants. *Cogn. Dev.* 6 355–375 10.1016/0885-2014(91)90044-E

[B149] Russell J. A. (1991). Culture and the categorization of emotions. *Psychol. Bull.* 110 426–450 10.1037/0033-2909.110.3.426 1758918

[B150] Russell J. A. (2003). Core affect and the psychological construction of emotion. *Psychol. Rev.* 110 145 10.1037/0033-295X.110.1.145 12529060

[B151] Russell J. A. Widen S. C. (2002). A label superiority effect in children’s categorization of facial expressions. *Soc. Dev.* 11 30–52 10.1111/1467-9507.00185

[B152] Saarni C. (1999). *The Development of Emotional Competence*. New York: Guilford.

[B153] Saffran J. R. Aslin R. N. Newport E. L. (1996). Statistical learning by 8-month-old infants. *Science* 274 1926–1928 10.1126/science.274.5294.1926 8943209

[B154] Schyns P. G. Goldstone R. L. Thibaut J. P. (1998). The development of features in object concepts. *Behav. Brain Sci.* 21 1–17 10.1017/S0140525X98000107 10097010

[B155] Shariff A. F. Tracy J. L. (2011). What are Emotion Expressions for? *Curr. Dir. Psychol. Sci.* 20 395–399 10.1177/0963721411424739

[B156] Shaver P. Schwartz J. Kirson D. O’Connor C. (1987). Emotion knowledge: further exploration of a prototype approach. *J. Pers. Soc. Psychol.* 52 1061–1086 10.1037/0022-3514.52.6.1061 3598857

[B157] Shukla M. White K. S. Aslin R. N. (2011). Prosody guides the rapid mapping of auditory word forms onto visual objects in 6-mo-old infants. *Proc. Natl. Acad. Sci. U.S.A.* 108 6038–6043 10.1073/pnas.1017617108 21444800 PMC3076873

[B158] Simcox T. Pilotti M. Mahamane S. Romero E. (2012). Does the language in which aversive stimulit are presented affect their processing? *Int. J. Biling.* 16 419–427 10.1177/1367006911425821

[B159] Sirois S. Spratling M. Thomas M. S. C. Westermann G. Mareschal D. Johnson M. H. (2008). Précis of neuroconstructivism: how the brain constructs cognition. *Behav. Brain Sci.* 31 321–331 10.1017/S0140525X0800407X 18578929

[B160] Sprengelmeyer R. Young A. W. Calder A. J. Karnat A. Lange H. Homberg V. (1996). Loss of disgust: perception of faces and emotions in Huntington’s disease. *Brain* 119 1647–1665 10.1093/brain/119.5.1647 8931587

[B161] Stanfield R. Zwaan R. (2001). The effect of implied orientation derived from verbal context on picture recognition. *Psychol. Sci.* 12 153–156 10.1111/1467-9280.00326 11340925

[B162] Steels L. Belpaeme T. (2005). Coordinating perceptually grounded categories through language: a case study for colour. *Behav. Brain Sci.* 28 469–489 10.1017/S0140525X05000087 16209771

[B163] Teinonen T. Fellman V. Näätänen R. Alku P. Huotilainen M. (2009). Statistical language learning in neonates revealed by event-related brain potentials. *BMC Neurosci.* 10:21 10.1186/1471-2202-10-21 PMC2670827 19284661

[B164] Thiessen E. D. Saffran J. R. (2003). When cues collide: use of stress and statistical cues to word boundaries by 7- to 9-month-old infants. *Dev. Psychol.* 39 706–716 10.1037/0012-1649.39.4.706 12859124

[B165] Tian X. Huber D. E. (2010). Testing an associative account of semantic satiation. *Cogn. Psychol.* 60 267–290 10.1016/j.cogpsych.2010.01.003 20156620 PMC2882703

[B166] Tominaga T. Choi H. Nagoshi Y. Wada Y. Fukui K. (2014). Relationship between alexithymia and coping strategies in patients with somatoform disorder. *Neuropsychiatr. Dis. Treat.* 10 55–62 10.2147/NDT.S55956 24403835 PMC3883553

[B167] Tomkins S. S. (1962). *Affect Imagery Consciousness - Volume II the Negative Affects*. Berlin: Springer Publishing Company.

[B168] Touroutoglou A. Lindquist K. A. Dickerson B. C. Barrett L. F. (in press). Intrinsic connectivity in the human brain does not reveal networks for “basic” emotions. *Soc. Cogn. Affect. Neurosci.* 10.1093/scan/nsv013 [Epub ahead of print]. PMC4560947 25680990

[B169] Vigliocco G. Meteyard L. Andrews M. Kousta S.-T. (2009). Toward a theory of semantic representation. *Lang. Cogn.* 1 219–247 10.1515/LANGCOG.2009.011

[B170] Widen S. C. (2013). Children’s interpretation of facial expressions: the long path from valence-based to specific discrete categories. *Emot. Rev.* 5 72–77 10.1177/1754073912451492

[B171] Widen S. C. Russell J. A. (2008). Children acquire emotion categories gradually. *Cogn. Dev.* 23 291–312 10.1016/j.cogdev.2008.01.002

[B172] Wierzbicka A. (2009). Language and metalanguage: key issues in emotion research. *Emot. Rev.* 1 3–14 10.1177/1754073908097175

[B173] Wilson T. D. Schooler J. W. (1991). Thinking too much: introspection can reduce the quality of preferences and decisions. *J. Pers. Soc. Psychol.* 60 331–339 10.1037/0022-3514.60.2.181 2016668

[B174] Wilson-Mendenhall C. D. Barrett L. F. Barsalou L. W. (2013a). Neural evidence that human emotions share core affective properties. *Psychol. Sci.* 24 947–956 10.1177/0956797612464242 23603916 PMC4015729

[B175] Wilson-Mendenhall C. D. Barrett L. F. Barsalou L. W. (2013b). Situating emotional experience. *Front. Hum. Neurosci.* 7:764 10.3389/fnhum.2013.00764 PMC3840899 24324420

[B176] Wilson-Mendenhall C. D. Barrett L. F. Simmons W. K. Barsalou L. W. (2011). Grounding emotion in situated conceptualization. *Neuropsychologia* 49 1105–1127 10.1016/j.neuropsychologia.2010.12.032 21192959 PMC3078178

[B177] Winawer J. Witthoft N. Frank M. C. Wu L. Wade A. R. Boroditsky L. (2007). Russian blues reveal effects of language on color discrimination. *Proc. Natl. Acad. Sci. U.S.A.* 104 7780–7785 10.1073/pnas.0701644104 17470790 PMC1876524

[B178] Xu F. (2002). The role of language in acquiring object kind concepts in infancy. *Cognition* 85 223–250 10.1016/S0010-0277(02)00109-9 12169410

[B179] Xu F. Cote M. Baker A. (2005). Labeling guides object individuation in 12-month-old infants. *Psychol. Sci.* 16 372–377 10.1111/j.0956-7976.2005.01543.x 15869696

[B180] Xu F. Griffiths T. L. (2011). Probabilistic models of cognitive development: towards a rational constructivist approach to the study of learning and development. *Cognition* 120 299–301 10.1016/j.cognition.2011.06.008 21723437

[B181] Xu F. Kushnir T. (2013). Infants are rational constructivist learners. *Curr. Dir. Psychol. Sci.* 22 28–32 10.1177/0963721412469396

[B182] Yehuda N. (2005). The language of dissociation. *J. Trauma Dissociation* 6 9–29 10.1300/J229v06n01_02 16150683

[B183] Zaki L. F. Coifman K. G. Rafaeli E. Berenson K. R. Downey G. (2013). Emotion differentiation as a protective factor against nonsuicidal self-injury in borderline personality disorder. *Behav. Ther.* 44 529–540 10.1016/j.beth.2013.04.008 23768678

[B184] Zinck A. Newen A. (2007). Classifying emotion: a developmental account. *Synthese* 161 1–25 10.1007/s11229-006-9149-2

[B185] Zunhammer M. Eberle H. Eichhammer P. Busch V. (2013). Somatic symptoms evoked by exam stress in university students: the role of alexithymia, neuroticism, anxiety and depression. *PLoS ONE* 8:e84911 10.1371/journal.pone.0084911 PMC3867544 24367700

[B186] Zwaan R. Stanfield R. Yaxley R. H. (2002). Language comprehenders mentally represent the shapes of objects. *Psychol. Sci.* 13 168–171 10.1111/1467-9280.00430 11934002

